# Supplementary material for: Autoimmune Th17 Cells Induced Synovial Stromal and Innate Lymphoid Cell Secretion of the Cytokine GM-CSF to Initiate and Augment Autoimmune Arthritis
Source: Immunity. 2018 Jun 19;48(6):1220–1232.e5. doi: 10.1016/j.immuni.2018.04.009 (PMC6024031; doi:10.1016/j.immuni.2018.04.009)
Supplement: Document S1. Figures S1–S4 [file mmc1.pdf]

Immunity, Volume *48*

Supplemental Information

Autoimmune Th17 Cells Induced Synovial Stromal and Innate Lymphoid Cell Secretion of the Cytokine GM-CSF to Initiate and Augment Autoimmune Arthritis

Keiji Hirota, Motomu Hashimoto, Yoshinaga Ito, Mayumi Matsuura, Hiromu Ito, Masao Tanaka, Hitomi Watanabe, Gen Kondoh, Atsushi Tanaka, Keiko Yasuda, Manfred Kopf, Alexandre J. Potocnik, Brigitta Stockinger, Noriko Sakaguchi, and Shimon Sakaguchi

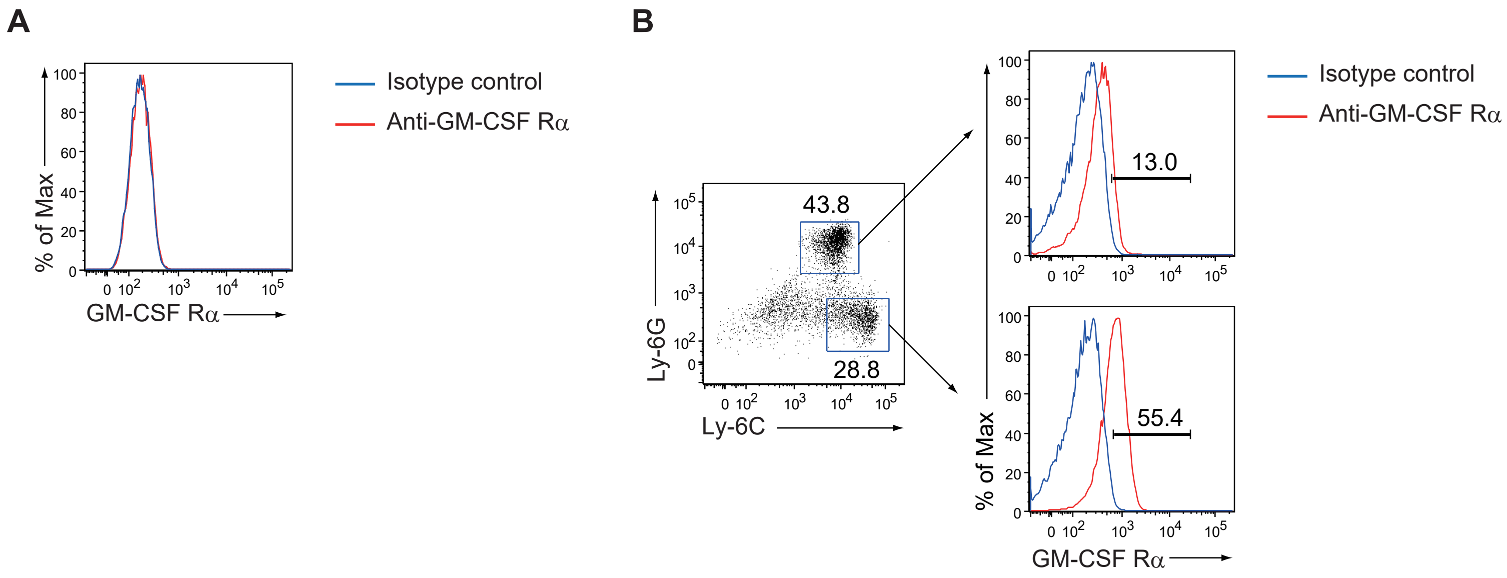

Figure S1. Flow cytometry of FLS and CD11b⁺ myeloid cells in inflamed joints, Related to Figure 3. **(A)** Staining for surface GM-CSF Rα of FLS isolated from SKG arthritic joints. **(B)** Surface GM-CSF Rα staining of $CD11b^+$ $Ly\text{-}6C^+$ $Ly\text{-}6G^-$ cells (inflammatory monocytes) and $CD11b^+$ $Ly\text{-}6C^+$ $Ly\text{-}6G^+$ cells (neutrophils) in SKG arthritic joints. Data are representative of two independent experiments.

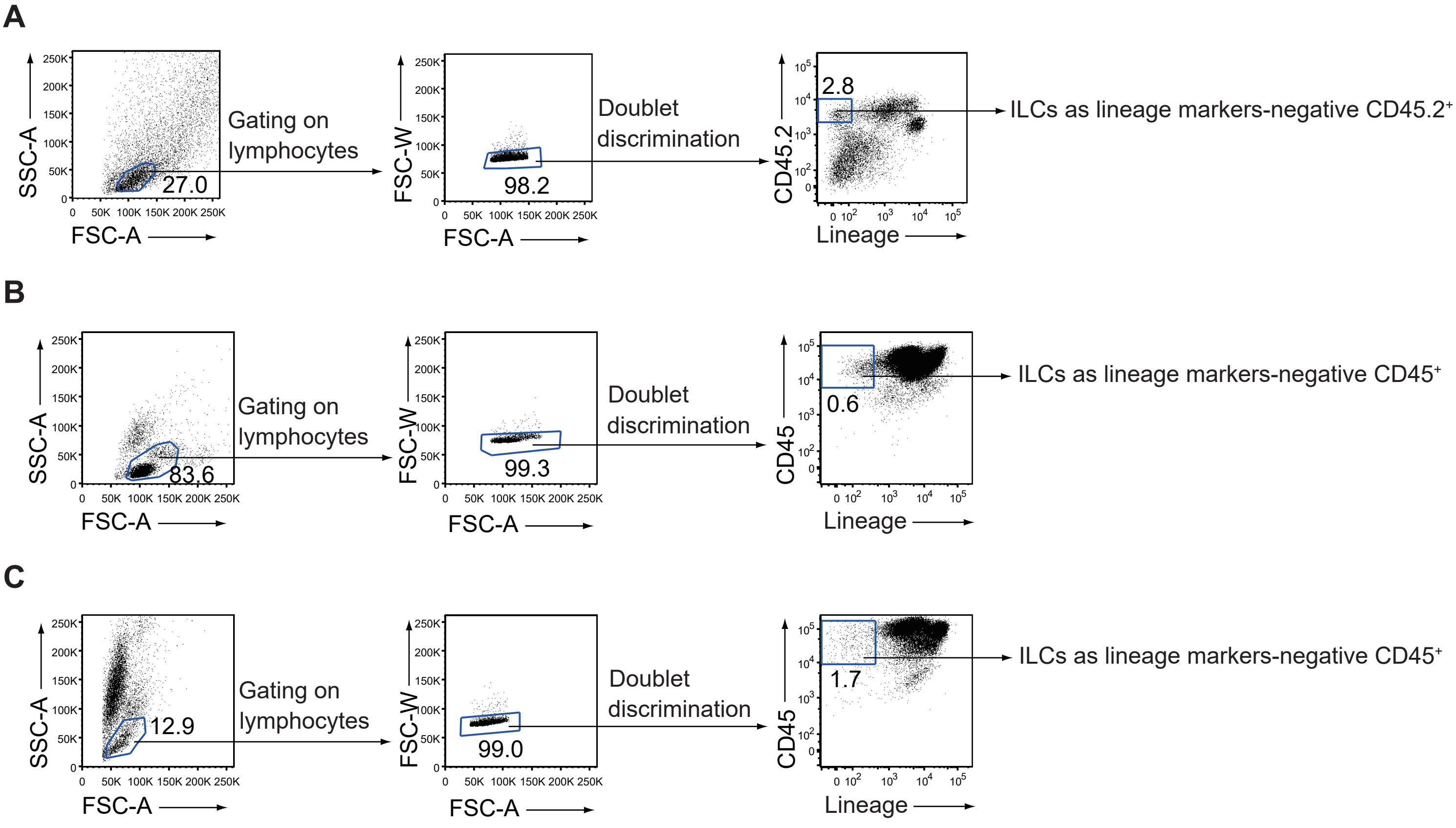

Figure S2. Gating strategies for mouse and human ILCs, Related to Figure 4 and Figure 7. Synovial cells from inflamed joints in mice **(A)**, peripheral blood **(B)** or synovial fluid **(C)** of RA patients were first gated on the lymphocyte fraction (left panel), then excluded of doublet cells using FSC-W (middle panel), and analyzed or purified as ILCs as CD45+ lineage markers-negative cells (right panel).

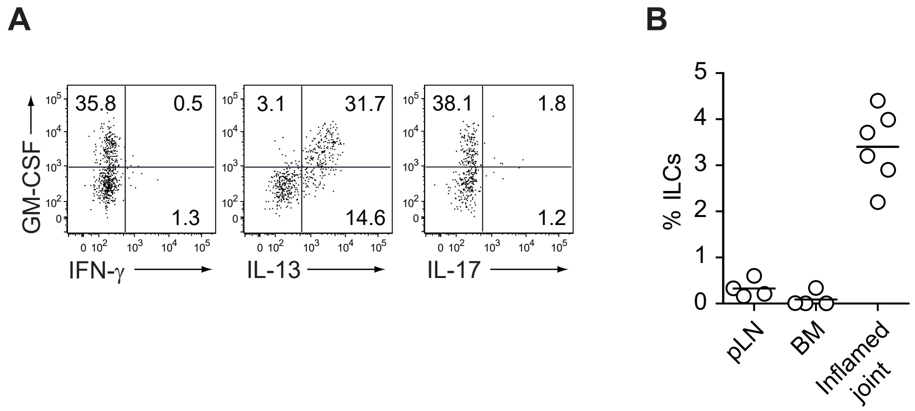

Figure S3. Flow cytometry of synovial ILCs in healthy joints of SKG mice, Related to Figure 4. **(A)** Flow cytometric analysis of intracellular GM-CSF, IFN-γ, IL-13, and IL-17 in ILCs from healthy joints of SKG mice. Data are representative of three independent experiments. **(B)** Proportion of ILCs in popliteal LNs (pLN), bone marrow (BM), and inflamed joints of SKG mice with severe arthritis induced by an injection of 20 mg mannan. Symbols represent individual mice. Horizontal lines indicate the means. Data are pooled from two independent experiments.

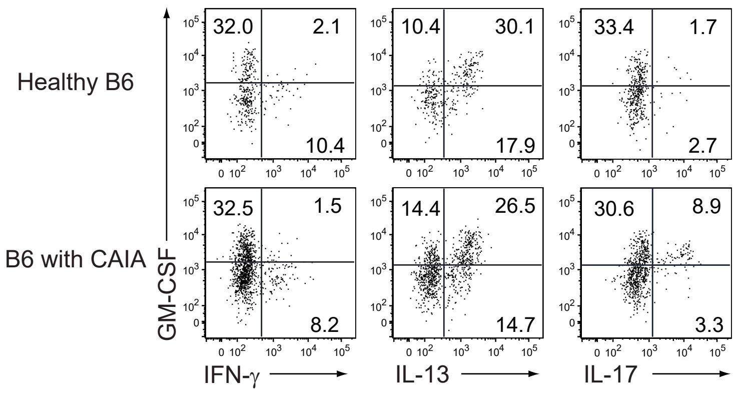

Figure S4. Flow cytometry of synovial ILCs in inflamed joints of C57/BL6 mice with collagen antibody-induced arthritis (CAIA), Related to Figure 4.

Flow cytometric analysis of intracellular GM-CSF, IFN-γ, IL-13, and IL-17 in ILCs from healthy or inflamed joints of C57/BL6 (B6) mice with CAIA. B6 mice were i.v. injected with a combination of monoclonal antibody cocktail (2 mg) specific for type II collagen (Arthrogen-CIA Arthritogenic Monoclonal Antibody, Chondrex, Inc.), i.p. injected with LPS (50 μg) three days later, and analyzed 2 weeks later. Data are representative of two independent experiments.
