# Supplementary material for: Autoimmune Th17 Cells Induced Synovial Stromal and Innate Lymphoid Cell Secretion of the Cytokine GM-CSF to Initiate and Augment Autoimmune Arthritis
Source: Immunity. 2018 Jun 19;48(6):1220–1232.e5. doi: 10.1016/j.immuni.2018.04.009 (PMC6024031; doi:10.1016/j.immuni.2018.04.009)
Supplement: Document S2. Article plus Supplemental Information [file mmc2.pdf]

Immunity

Article

Autoimmune Th17 Cells Induced Synovial Stromal and Innate Lymphoid Cell Secretion of the Cytokine GM-CSF to Initiate and Augment Autoimmune Arthritis

Graphical Abstract

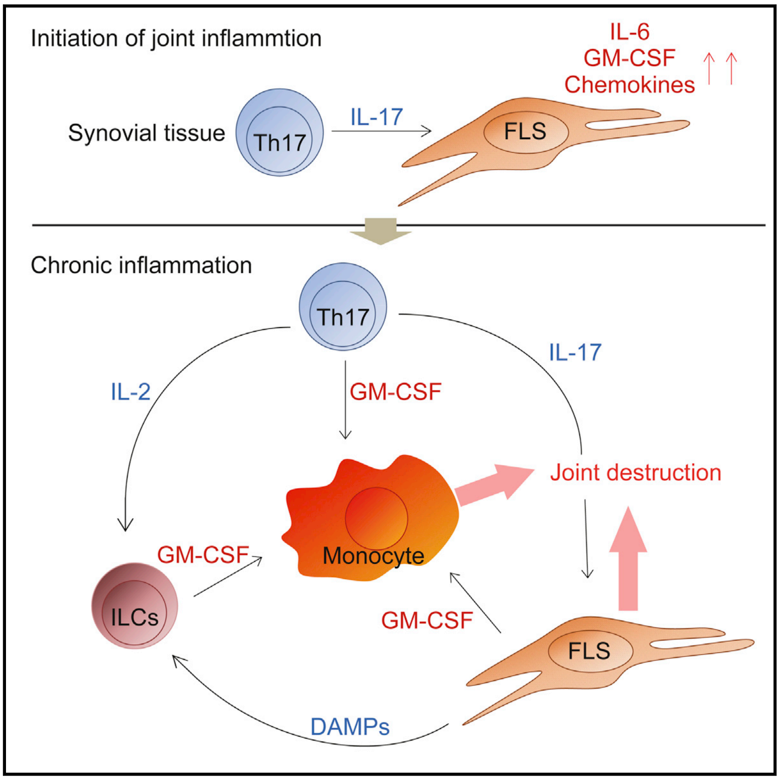

Authors

Keiji Hirota, Motomu Hashimoto, Yoshinaga Ito, ..., Brigitta Stockinger, Noriko Sakaguchi, Shimon Sakaguchi

Correspondence

hkeiji@infront.kyoto-u.ac.jp (K.H.), shimon@ifrec.osaka-u.ac.jp (S.S.)

In Brief

It remains obscure how joint inflammation in rheumatoid arthritis is initiated and progressing. In this study, Hirota et al. identified in an animal model of rheumatoid arthritis an inflammatory cellular cascade instigated by an arthritogenic T helper subset and enhanced by GM-CSF-producing synovial-resident innate lymphoid cells.

Highlights

- T cell production of GM-CSF is dispensable for the initiation of arthritis
- GM-CSF from stromal cells is crucial for the initiation of autoimmune arthritis
- GM-CSF-producing synovial-resident ILCs augment autoimmune arthritis
- ILC production of GM-CSF is stimulated by IL-2, IL-33, or TLR9 ligands

Hirota et al., 2018, Immunity *48*, 1220–1232
June 19, 2018 © 2018 Elsevier Inc.
https://doi.org/10.1016/j.immuni.2018.04.009

Immunity
Article

Autoimmune Th17 Cells Induced Synovial Stromal and Innate Lymphoid Cell Secretion of the Cytokine GM-CSF to Initiate and Augment Autoimmune Arthritis

Keiji Hirota,[1,5,*] Motomu Hashimoto,[2,9] Yoshinaga Ito,[3,9] Mayumi Matsuura,[3] Hiromu Ito,[2,4] Masao Tanaka,[2] Hitomi Watanabe,[5] Gen Kondoh,[5] Atsushi Tanaka,[1] Keiko Yasuda,[1] Manfred Kopf,[6] Alexandre J. Potocnik,[7] Brigitta Stockinger,[8] Noriko Sakaguchi,[1] and Shimon Sakaguchi[1,3,10,*]
[1]Department of Experimental Immunology, Immunology Frontier Research Center, Osaka University, Osaka 565-0871, Japan
[2]Department of Advanced Medicine for Rheumatic Diseases, Graduate School of Medicine, Kyoto University, Kyoto 606-8507, Japan
[3]Laboratory of Experimental Immunology, Institute for Frontier Life and Medical Sciences, Kyoto University, Kyoto 606-8507, Japan
[4]Department of Orthopedic Surgery, Graduate School of Medicine, Kyoto University, Kyoto 606-8507, Japan
[5]Laboratory of Integrative Biological Science, Institute for Frontier Life and Medical Sciences, Kyoto University, Kyoto 606-8507, Japan
[6]Department of Biology, Institute of Molecular Health Sciences, ETH Zürich, 8093 Zürich, Switzerland
[7]Institute of Immunology and Infection Research, The University of Edinburgh, Edinburgh EH9 3FL, UK
[8]AhR Immunity Laboratory, The Francis Crick Institute, London NW1 1AT, UK
[9]These authors contributed equally
[10]Lead Contact
*Correspondence: hkeiji@infront.kyoto-u.ac.jp (K.H.), shimon@ifrec.osaka-u.ac.jp (S.S.)
https://doi.org/10.1016/j.immuni.2018.04.009

SUMMARY

Despite the importance of Th17 cells in autoimmune diseases, it remains unclear how they control other inflammatory cells in autoimmune tissue damage. Using a model of spontaneous autoimmune arthritis, we showed that arthritogenic Th17 cells stimulated fibroblast-like synoviocytes via interleukin-17 (IL-17) to secrete the cytokine GM-CSF and also expanded synovial-resident innate lymphoid cells (ILCs) in inflamed joints. Activated synovial ILCs, which expressed CD25, IL-33Ra, and TLR9, produced abundant GM-CSF upon stimulation by IL-2, IL-33, or CpG DNA. Loss of GM-CSF production by either ILCs or radio-resistant stromal cells prevented Th17 cell-mediated arthritis. GM-CSF production by Th17 cells augmented chronic inflammation but was dispensable for the initiation of arthritis. We showed that GM-CSF-producing ILCs were present in inflamed joints of rheumatoid arthritis patients. Thus, a cellular cascade of autoimmune Th17 cells, ILCs, and stromal cells, via IL-17 and GM-CSF, mediates chronic joint inflammation and can be a target for therapeutic intervention.

INTRODUCTION

Proinflammatory cytokines such as IL-1, IL-6, IL-17, IL-23, GM-CSF, and TNF-α are important in the development and maintenance of chronic inflammatory disorders such as autoimmune disease. Neutralization of these cytokines or blockade of their receptors is effective in hampering the progression of tissue inflammation and inducing long-term remission in autoimmune disorders (Cho and Feldman, 2015). Recent genome-wide association studies of immune-mediated diseases have revealed common inflammatory pathways, involving the genes encoding these cytokines and receptors, in various autoimmune diseases (Parkes et al., 2013). It remains to be determined, however, how key inflammatory cytokines control non-lymphoid as well as lymphoid target cells in autoimmune tissue inflammation, how environmental factors, as well as genetic factors, contribute to the inflammation, and how the cytokine-dependent inflammatory pathways can be targeted to treat or prevent autoimmune diseases.

IL-17-producing T helper (Th17) cells play critical roles for host defense against infectious pathogens but can also mediate various autoimmune or inflammatory reactions. They express the lineage defining transcription factor Rorγt and require IL-6 and TGF-β for their differentiation and IL-1 and IL-23 for their terminal effector functions (Ivanov et al., 2006; McGeachy et al., 2009; Veldhoen et al., 2006). In rheumatoid arthritis (RA), for example, it has been shown that various inflammatory cytokines including TNF-α, IL-1, and IL-6 are involved in joint inflammation and that T cells interact with tissue-resident macrophage-like or fibroblast-like synoviocytes (FLSs) in destroying cartilage and bone in the joint (Bartok and Firestein, 2010). It still remains unclear, however, how arthritogenic Th17 cells mediate chronic tissue inflammation in the joint via local cytokine and cellular networks.

GM-CSF is a key proinflammatory cytokine for the activation of dendritic cells (DCs) and macrophages; for example, DCs respond to GM-CSF to secrete IL-6 and IL-23, which sustain pathogenic Th17 cells *in vivo* (Sonderegger et al., 2008). Moreover, IL-1 and IL-23 signaling drives Rorγt-expressing Th17 cells to secrete GM-CSF, perpetuating autoimmune inflammation, for example, in mouse experimental autoimmune encephalomyelitis (EAE) (Codarri et al., 2011; El-Behi et al., 2011). The antigen-presentation capacity of monocytes and synovial

 © 2018 Elsevier Inc.

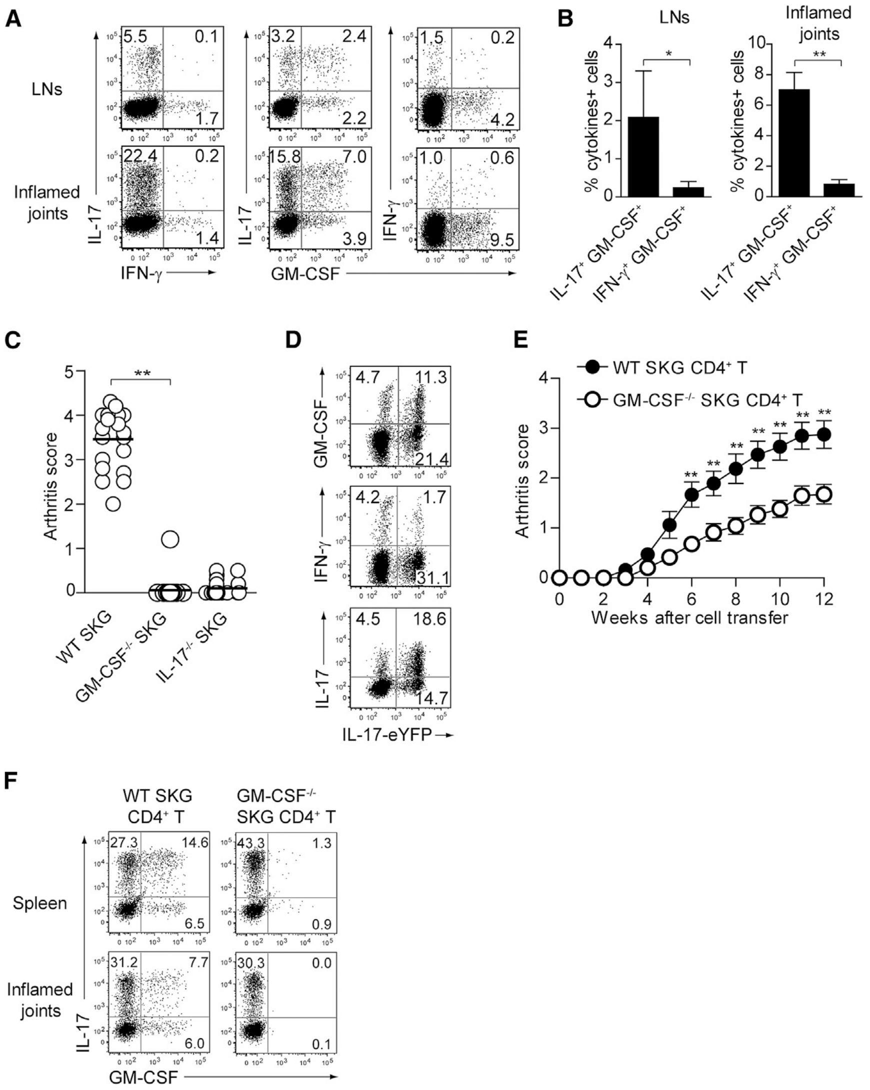

Figure 1. GM-CSF-Producing T Helper Cells Are Dispensable for GM-CSF-Dependent Autoimmune Arthritis Development

(A) Intracellular IL-17, IFN-γ, and GM-CSF staining of $CD4^+$ T cells from popliteal LNs or inflamed joints.

(B) Proportion of cytokine-producing cells in $CD4^+$ T cells from individual mice as shown in (A). Vertical bars mean SD (n = 3).

(C) Arthritis scores assessed in individual SKG, *Csf2*$^{-/-}$ SKG, or *Il17a*$^{-/-}$ SKG mice (n = 20 each) 3 months after single i.p. injection of 20 mg mannan.

(D) Intracellular IL-17, IFN-γ, and GM-CSF staining of $CD4^+$ T cells from inflamed joints of *Il17a*^{Cre} R26R^{eYFP} SKG mice.

(E) Arthritis development after adoptive transfer of $CD4^+$ T cells from WT or *Csf2*$^{-/-}$ SKG mice into *Rag2*$^{-/-}$ mice (n = 17 each, SEM). The severity of arthritis was monitored every week after transfer of 1×10^6 $CD4^+$ T cells.

(F) Intracellular IL-17 and GM-CSF staining of $CD4^+$ T cells from spleens and inflamed joints of *Rag2*$^{-/-}$ mice with $CD4^+$ T cells transfer as shown in (E).

*$p < 0.05$, **$p < 0.01$. Data are representative of three independent experiments (A, B, D, and F) or pooled from three experiments (C and E).

inflammatory macrophages can also be enhanced by stimulation with GM-CSF through upregulation of MHC class II expression (Alvaro-Gracia et al., 1989). In addition, GM-CSF signaling evokes an inflammatory signature in $CCR2^+Ly6C^{hi}$ monocytes and drives them to induce tissue damage (Croxford et al., 2015). GM-CSF thus appears to possess pleiotropic effects on monocytes and/or DCs and Th17 cells, augmenting the activation of innate and adaptive immune cells and amplifying tissue inflammation.

The SKG strain of mice, carrying a point mutation in the gene encoding the T cell receptor (TCR)-proximal signaling molecule ZAP-70, develops $CD4^+$ T cell-mediated autoimmune arthritis, which clinically and immunologically resembles RA in humans (Hata et al., 2004; Sakaguchi et al., 2003). The mice spontaneously develop the disease in a microbially conventional environment but not under a specific-pathogen-free (SPF) condition. Yet the disease can be induced in SPF SKG mice by stimulation of innate immunity via Toll-like receptors (TLRs), the Dectin pathway, or complement activation pathways (Hashimoto et al., 2010; Yoshitomi et al., 2005). We previously demonstrated, by using SKG mice, how self-reactive T cells are generated in the process of thymic-positive and -negative selection (Sakaguchi et al., 2003), become activated in the periphery by recognizing self-antigens, differentiate into arthritogenic Th17 cells upon stimulation of innate immunity (Hirota et al., 2007a), migrate into the joints (Hirota et al., 2007b), and aggress self-antigens expressed by synoviocytes (Ito et al., 2014). In addition, dysfunction of $Foxp3^+$ regulatory T cells due to the ZAP-70 mutation facilitates autoimmune T cells to expand, become activated, and exert their effector functions, causing autoimmune diseases in a wide spectrum of organs or tissues (Tanaka et al., 2010). These features make this spontaneous model of autoimmune arthritis suitable for elucidating how Th17 cells mediate autoimmune diseases, especially RA, via interacting with other lymphoid and non-lymphoid cells at the inflammation site and controlling their production of inflammatory cytokines.

In this report, we showed via the SKG model of autoimmune arthritis that arthritogenic Th17 cells orchestrated the progression of chronic joint inflammation by stimulating radio-resistant stromal cells including FLSs to secrete GM-CSF and subsequently by expanding GM-CSF-producing innate lymphoid cells (ILCs). Notably, GM-CSF secretion from ILCs was regulated by IL-2, the alarmin IL-33, and endogenous TLR-9 ligands released from damaged tissue-resident cells, in inflamed joints. The results demonstrate how antigen-specific self-reactive T cells stimulate the local cellular and cytokine networks that drive chronic tissue inflammation.

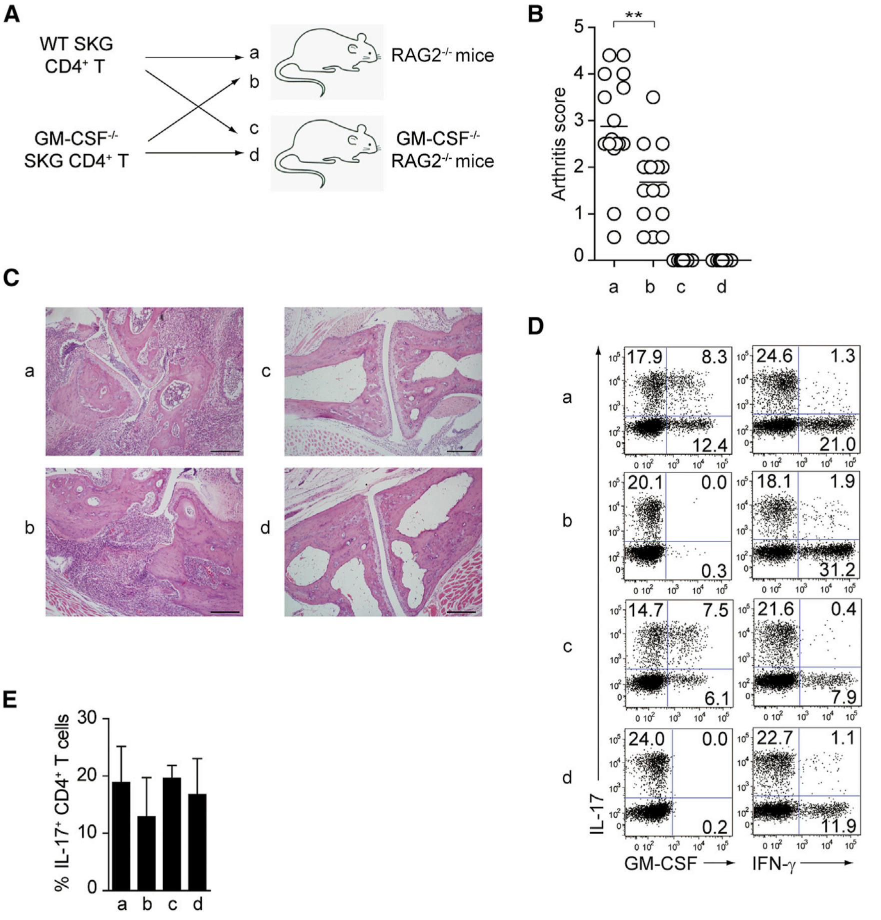

Figure 2. GM-CSF from Non-T Cells Is Crucial for the Initiation of Autoimmune Arthritis

(A) Experimental design of adoptive transfer of $CD4^+$ T cells from WT or *Csf2*$^{-/-}$ SKG mice into *Rag2*$^{-/-}$ or *Csf2*$^{-/-}$*Rag2*$^{-/-}$ mice. Arthritis scores of four groups (a–d) of mice were assessed 3 months after transfer of 1×10^6 $CD4^+$ T cells.

(B) Arthritis scores of the four groups mice (n = 15 or 16 each) shown in (A). Horizontal bars indicate the means.

(C) Representative joint histology of the groups shown in (A). Scale bars indicate 200 μm.

(D) Flow cytometry of splenic $CD4^+$ T cells stained for intracellular IL-17 and GM-CSF or IFN-γ.

(E) Proportion of IL-17-producing $CD4^+$ T cells from individual mice as shown in (D). Vertical bars mean SD (n = 3).

$^{**}p < 0.01$. Data are representative of three independent experiments (C–E) or pooled from three independent experiments (B).

RESULTS

GM-CSF as a Crucial Inflammatory Mediator of Autoimmune Arthritis

A single injection of 20 mg mannan, an activator of the lectin pathway for complement activation, is able to synchronously evoke T cell-mediated autoimmune arthritis within 2–3 weeks in SPF SKG mice with an increase in Th17 cells in lymph nodes and joints (Hashimoto et al., 2010). In the draining lymph nodes and inflamed joints of mannan-treated SKG mice, approximately 2% and 7%, respectively, of $CD4^+$ T cells co-expressed IL-17 and GM-CSF, but not IFN-γ (Figures 1A and 1B). In addition, GM-CSF (encoded by *Csf2*)-deficient (*Csf2*$^{-/-}$) SKG mice were highly resistant to the induction of autoimmune arthritis by mannan as were *Il17a*$^{-/-}$ SKG mice, indicating a crucial role of GM-CSF for arthritis development in SKG mice (Figure 1C; Hirota et al., 2007a).

To determine the origin of such GM-CSF-producing cells in arthritic SKG mice, we generated IL-17-fate reporter SKG mice by crossing SKG mice with *Il17a*^{Cre} and R26R^{eYFP} fate reporter strains (Hirota et al., 2011). Following mannan treatment, more than 30% of $CD4^+$ T cells in inflamed joints were $eYFP^+$, indicating that they were producing IL-17 or had once produced the cytokine (exTh17 cells) (Figure 1D). In addition, one-third of $eYFP^+$ cells were producing GM-CSF, indicating that IL-17-producing $CD4^+$ T cells produced GM-CSF in inflamed joints. Also, only ∼5% of $eYFP^+$ cells were producing IFN-γ, suggesting that differentiation toward Th1-like cells was not the main cell fate of Th17 or exTh17 cells in this model, in contrast with EAE, in which the vast majority of exTh17 cells were producing IFN-γ (Hirota et al., 2011).

Next, we adoptively transferred $CD4^+$ T cells from *Csf2*$^{-/-}$ or wild-type (WT) SKG mice into *Rag2*$^{-/-}$ mice to determine the pathogenicity of GM-CSF-producing $CD4^+$ T cells (Figure 1E). *Csf2*$^{-/-}$ SKG $CD4^+$ T cells were able to induce autoimmune arthritis in all the recipient mice, although arthritis was significantly less severe than that induced by WT SKG $CD4^+$ T cell transfer. Analysis of IL-17 and GM-CSF production by $CD4^+$ T cells in the spleen and inflamed joints revealed that IL-17-producing $CD4^+$ T cells, which were crucial for initiating SKG autoimmune arthritis (Hirota et al., 2007a), had similarly differentiated from *Csf2*$^{-/-}$ or WT SKG $CD4^+$ T cells (Figure 1F).

These results demonstrated that both T cell-derived and non-T cell-derived GM-CSF contributed to joint inflammation in SKG mice and that GM-CSF from Th cells was not mandatory for this induction of autoimmune arthritis.

GM-CSF Secreted from Non-T Cells Is Crucial for Initiating Autoimmune Arthritis

To further assess the possible contribution of GM-CSF from non-CD4 T cells to arthritis development, we transferred *Csf2*$^{-/-}$ or WT SKG $CD4^+$ T cells into *Rag2*$^{-/-}$ or *Csf2*$^{-/-}$*Rag2*$^{-/-}$ mice (Figure 2A). Transfer of *Csf2*$^{-/-}$ SKG $CD4^+$ T cells induced histologically evident arthritis in *Rag2*$^{-/-}$ mice although the arthritis was macroscopically less severe than WT SKG $CD4^+$ T cell transfer (Figures 2B and 2C). In contrast, not only *Csf2*$^{-/-}$ SKG $CD4^+$ T cells but also WT SKG $CD4^+$ T cells completely failed to induce arthritis macroscopically and histologically in *Csf2*$^{-/-}$*Rag2*$^{-/-}$ mice. Intracellular cytokine staining of transferred *Csf2*$^{-/-}$ or WT SKG $CD4^+$ T cells revealed that both populations were activated

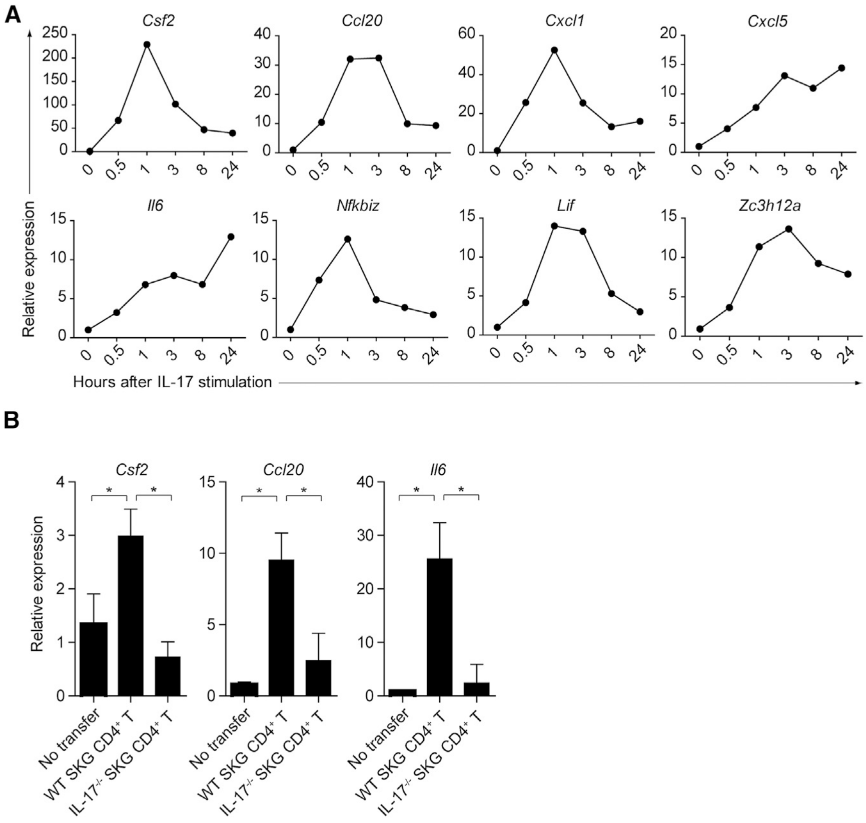

Figure 3. Induction of GM-CSF in FLSs Stimulated with IL-17

(A) Quantitative RT-PCR analysis for the expression of designated genes in IL-17-stimulated FLSs. FLSs (2.5 × 10^4) were stimulated with 50 ng/mL rmIL-17 and harvested at the indicated time points. mRNA expression is presented relative to the expression of *Hprt1*.

(B) Quantitative RT-PCR analysis for the expression of designated genes in synoviocytes from *Rag2*$^{-/-}$ mice with CD4$^+$ T cell transfer. CD45$^-$Podoplanin$^+$ synoviocytes (3 × 10^4) were sorted from inflamed joints of *Rag2*$^{-/-}$ mice 4 weeks after transfer of 1 × 10^6 SKG or *Il17a*$^{-/-}$ SKG CD4$^+$ T cells. Vertical bars mean SD (n = 3).

Data are representative of two independent experiments.

and had differentiated into IL-17-producing CD4$^+$ T cells regardless of whether the hosts produced GM-CSF or not (Figures 2D and 2E). Thus, the failure in arthritis development following transfer of CD4$^+$ T cells into *Csf2*$^{-/-}$ hosts could be attributed not to impaired Th17 cell differentiation but to impaired GM-CSF production by certain host non-T cells stimulated by Th17 cells.

FLSs Secrete GM-CSF in Response to IL-17

We next attempted to determine the non-T cell source of GM-CSF in inflamed joints in SKG mice. FLSs are known to be key effector cells capable of secreting large amounts of pro-inflammatory mediators (e.g., tissue-degrading enzymes and cytokines such as TNF-α), which destroy the cartilage and bone in SKG arthritis as in human RA (Bartok and Firestein, 2010; Hata et al., 2004). Gene expression analysis of FLSs that were freshly isolated from SKG arthritic joints and stimulated *in vitro* with recombinant IL-17 revealed quick upregulation within 1–3 hr of the expression of *Csf2*, *Ccl20*, *Cxcl1*, *Cxcl5*, *Il6*, *Nfkbiz*, *Lif*, and *Zc3h12a* (Figure 3A). Yet activated FLSs themselves neither expressed GM-CSF receptor-alpha nor responded to recombinant GM-CSF (Figure S1A and data not shown). The cells predominantly expressing GM-CSF receptor-alpha in inflamed joints were CD11b$^+$Ly-6C$^+$Ly-6G$^-$ inflammatory monocytes, as reported previously with EAE (Croxford et al., 2015), indicating that this population could be a main target of GM-CSF in the joint (Figure S1B). In addition, cell transfer of WT, but not *Il17a*$^{-/-}$, SKG CD4$^+$ T cells into *Rag2*$^{-/-}$ mice significantly induced *Csf2*, *Ccl20*, and *Il6* transcription in CD45$^-$podoplanin$^+$ synoviocytes that were purified from the joints 4 weeks after cell transfer (Figure 3B). Thus, one of the cellular targets of arthritogenic Th17 cells is FLSs, which secrete GM-CSF upon IL-17 stimulation.

GM-CSF-Producing Synovial-Resident ILCs Expand in Arthritic Joints and Augment Autoimmune Arthritis

Next, in order to search for a potential hematopoietic source of GM-CSF other than T cells in joint inflammation, we prepared single-cell suspensions from enzyme-digested inflamed joints in *Rag2*$^{-/-}$ mice that had received *Csf2*$^{-/-}$ or WT SKG CD4$^+$ T cells. By intracellular GM-CSF staining, CD4$^+$ and non-CD4$^+$ T cell populations in CD45$^+$ hematopoietic cells contained similar percentages of GM-CSF-producing cells in *Rag2*$^{-/-}$ mice transferred with WT SKG CD4$^+$ T cells, whereas non-CD4 cells were the only source of GM-CSF in those transferred with *Csf2*$^{-/-}$ SKG CD4$^+$ T cells (Figure 4A). To characterize the non-CD4 source of GM-CSF, various CD45$^+$ cell populations were purified from SKG inflamed joints by the use of cell lineage markers and assessed for their cytokine expression by quantitative RT-PCR (Figure 4B). The vast majority of joint-infiltrating cells were CD11b$^+$Ly-6G$^-$ inflammatory monocytes or macrophages and CD11b$^+$Ly-6G$^+$ neutrophils, a common feature of Th17 cell-mediated inflammation (Iwakura et al., 2011). However, these populations of neutrophils and inflammatory monocytes did not express *Csf2* while they specifically expressed *Il1b*. In contrast, lymphoid populations including CD4$^+$ T cells and lineage marker-negative ILCs expressed *Csf2* but not *Il1b*. 2% to 4% of CD45$^+$ lymphocytes in arthritic joints were lineage-negative CD45$^+$ ILCs, which were equivalent in ratio between *Rag2*$^{-/-}$ mice transferred with *Csf2*$^{-/-}$ or WT SKG CD4$^+$ T cells (Figures 4C, 4D, and S2A for gating strategy). We also found a fraction of synovial-resident ILCs in healthy joints of SKG mice (which are on the BALB/c background) and other mouse strains (Figures 4E, S3A, and S4). Expansion of these synovial ILCs expressing Ki-67 was detectable only in inflamed joints, but not in the draining LNs or bone marrow (BM), suggesting that synovial ILCs

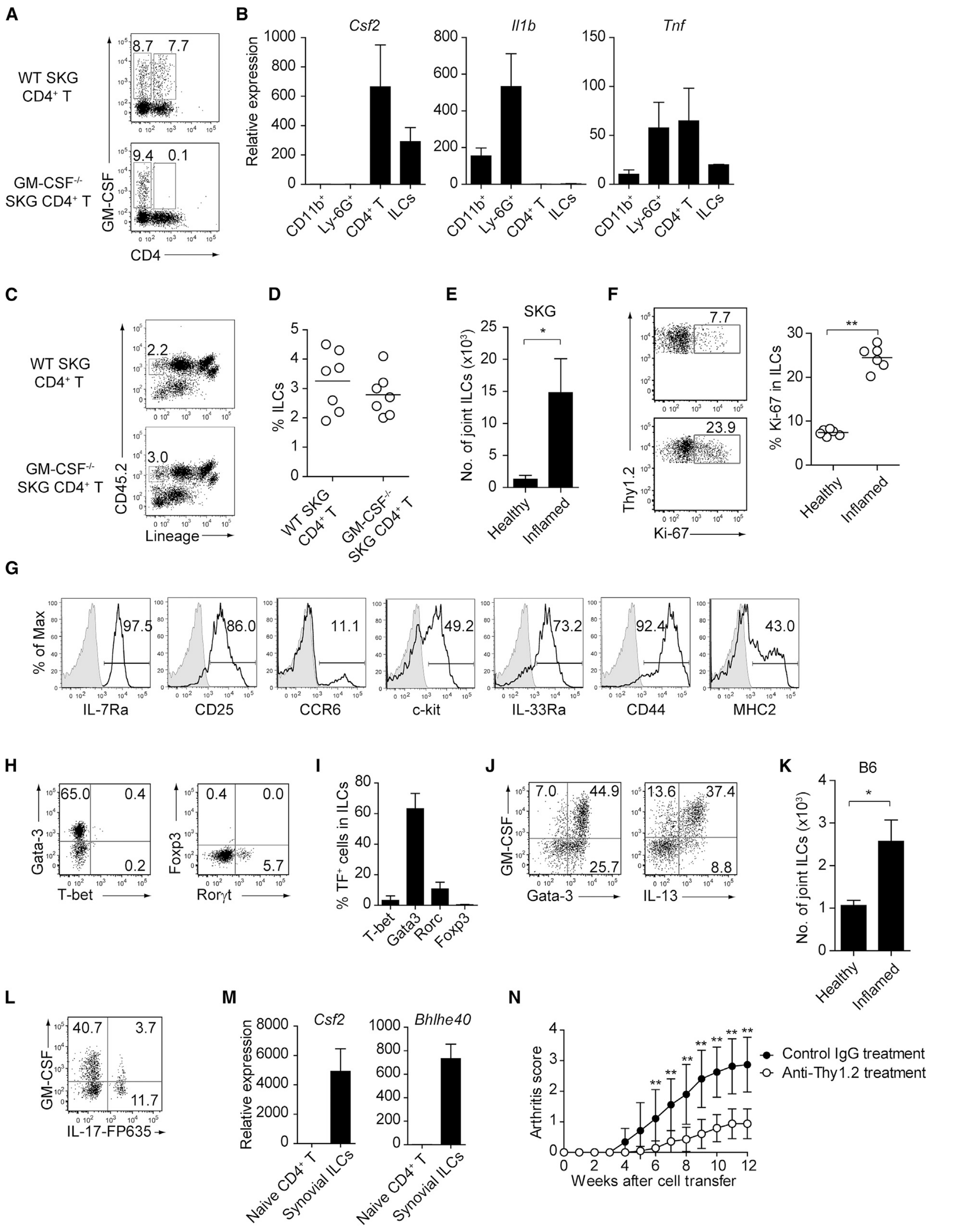

(legend on next page)

preferentially expanded in inflamed joints, but might scarcely migrate to the adjacent lymphoid organs (Figures 4E, 4F, and S3B).

By flow cytometry, most of the joint-infiltrating ILCs expressed IL-7Ra, CD25, IL-33Ra, and CD44, while nearly a half of them expressed c-kit and/or MHC class II, and ~10% of them expressed CCR6 (Figure 4G). Further analysis of transcription factors defining ILC subsets revealed that approximately 60% and 6% of the ILCs expressed Gata-3 or Rorγt, respectively, indicating predominant expansion of ILC2s in arthritic joints, as shown in intestinal infection (Figures 4H and 4I; Hoyler et al., 2012). Indeed, GM-CSF-producing ILCs in healthy and inflamed joints expressed Gata-3 and/or IL-13, which are the signature transcription factor and cytokine, respectively, of ILC2s (Figures 4J and S3A). ILCs with a similar phenotype were also present in normal joints and expanded in collagen antibody-induced arthritis in C57/BL6 mice, indicating that the presence and expansion of GM-CSF-producing synovial ILCs was not dependent on the mouse genetic background or the mode of arthritis induction by autoimmune Th17 cells or autoantibody (Figures 4K and S4). In addition, inflamed joints contained Rorγt-expressing ILC3s (Figure 4H). As ILC3s were reportedly able to produce GM-CSF in the intestine to maintain gut homeostasis (Mortha et al., 2014), we attempted with IL-17-fate reporter SKG mice to determine whether joint ILC3s, whose signature cytokine is IL-17, also produced GM-CSF. In correlation with the small percentage (~6%) of Rorγt-expressing ILC3s shown in Figure 4H, joint ILCs contained a small fraction (~4%) of IL-17-fate reporter-positive cells producing GM-CSF, suggesting that a part of GM-CSF-producing ILCs were derived from ILC3s in this model (Figure 4L). Synovial ILCs also highly expressed the transcription factor encoded by *Bhlhe40*, which reportedly controlled GM-CSF production by pathogenic Th cells (Figure 4M; Lin et al., 2014; Martínez-Llordella et al., 2013).

To further determine the contribution of these ILCs to arthritis development, we attempted to selectively deplete ILCs by anti-Thy1.2 mAb in Thy1.2$^+$ *Rag2*$^{-/-}$ mice that had been transferred with CD4$^+$ T cells from Thy1.1$^+$ congenic SKG mice (Figure 4N). Multiple injections of 500 μg anti-Thy1.2 mAb every week indeed reduced the severity of autoimmune arthritis significantly.

Taken together, synovial ILCs selectively expanded in arthritic joints, secreting GM-CSF and contributing to the development of autoimmune arthritis.

GM-CSF from Both Radio-Resistant Stromal Cells Including FLSs and ILCs Is Essential for Autoimmune Arthritis Development

To assess the contribution of GM-CSF from either stromal cells or ILCs to autoimmune arthritis, we x-irradiated (6Gy) *Rag2*$^{-/-}$ or *Csf2*$^{-/-}$*Rag2*$^{-/-}$ mice, reconstituted the mice with *Rag2*$^{-/-}$ or *Csf2*$^{-/-}$*Rag2*$^{-/-}$ BM cells, transferred to them *Csf2*$^{-/-}$ SKG CD4$^+$ T cells 6 weeks after BM reconstitution, and assessed arthritis score 12 weeks after CD4$^+$ T cell transfer (Figure 5A). *Rag2*$^{-/-}$ BM-transferred x-irradiated *Csf2*$^{-/-}$*Rag2*$^{-/-}$ mice and *Csf2*$^{-/-}$*Rag2*$^{-/-}$ BM-transferred x-irradiated *Rag2*$^{-/-}$ mice, in which GM-CSF production was restricted to ILCs or radio-resistant stromal cells including FLSs, respectively, developed significantly less severe arthritis than *Rag2*$^{-/-}$ BM-transferred x-irradiated *Rag2*$^{-/-}$ mice, in which both ILCs and radio-resistant stromal cells produced GM-CSF (Figure 5B). The percentage of total synovial ILCs expanding in inflamed joints was comparable between *Rag2*$^{-/-}$ BM transfer and *Csf2*$^{-/-}$*Rag2*$^{-/-}$ BM transfer, although the total number of synovial ILCs was smaller in the latter chimeras presumably because of less severe synovial inflammation (Figure 5C, data not shown). Also, the latter still possessed a small number of recipient-derived GM-CSF-producing ILCs (Figure 5D), indicating that reduction, if not complete abrogation, of GM-CSF production in ILCs was able to attenuate arthritis severity. In addition, by *Csf2*$^{-/-}$ BM transfer, the proportion of ILCs co-expressing GM-CSF and IL-13 decreased, with similar proportions of total IL-13$^+$ ILCs, suggesting that joint inflammation had expanded

Figure 4. GM-CSF-Producing ILCs in Inflamed Joints

(A) Flow cytometry analysis of GM-CSF expression by CD4$^+$ T cells and CD4$^-$ cells among CD45$^+$ joint infiltrating cells in *Rag2*$^{-/-}$ mice transferred with CD4$^+$ T cells from WT or *Csf2*$^{-/-}$ SKG mice.

(B) Quantitative RT-PCR analysis of *Csf2*, *Il1b*, and *Tnf* in CD11b$^+$Ly-6G$^-$ (CD11b$^+$), CD11b$^+$Ly-6G$^+$ (Ly-6G$^+$), CD4$^+$ T cells, and ILCs sorted from arthritic joints of mannan-treated SKG mice (n = 3). mRNA expression is presented relative to the expression of *Hprt1*.

(C) Flow cytometry of joint infiltrating cells in *Rag2*$^{-/-}$ mice transferred with CD4$^+$ T cells from WT or *Csf2*$^{-/-}$ SKG mice. Cells were stained for CD45.2 and lineage markers (a cocktail of CD3, CD4, CD8, CD11b, CD11c, CD19, and DX-5).

(D) Proportion of ILCs in *Rag2*$^{-/-}$ mice transferred with CD4$^+$ T cells as shown in (C). Each symbol represents an individual mouse. Horizontal bars indicate the means.

(E) Total cell number of ILCs from healthy or inflamed joints of SKG mice (n = 3).

(F) Flow cytometry of synovial ILCs (CD45.2$^+$ lineage markers-negative Thy1.2$^+$ cells as shown in C) for Ki-67 expression.

(G) Flow cytometry of synovial ILCs (CD45.2$^+$ lineage markers-negative Thy1.2$^+$ cells as shown in C) for cell surface expression of IL-7Ra, CD25, CCR6, c-kit, IL-33Ra, CD44, and MHC2.

(H) Flow cytometry of synovial ILCs (as shown in C) for intranuclear expression of the transcription factor T-bet, Gata-3, Rorγt, and Foxp3.

(I) Proportion of the transcription factor-expressing synovial ILCs (n = 3) as shown in (H).

(J) Flow cytometry of synovial ILCs (as shown in C) for the expression of GM-CSF, Gata-3, and IL-13.

(K) Total cell numbers of ILCs from healthy or inflamed joints of C57/BL6 (B6) mice with collagen antibody-induced arthritis (n = 3). Data are representative of two independent experiments.

(L) Flow cytometry of synovial ILCs for the expression of GM-CSF and FP635 in arthritic *Il17a*^{Cre} R26R^{FP635} SKG mice.

(M) Quantitative RT-PCR analysis of *Csf2* and *Bhlhe40* in splenic naive CD25$^-$CD44^{lo}CD4$^+$ T cells (naive CD4$^+$ T) and synovial ILCs (n = 3) as shown in (C).

(N) The effects of ILC depletion on arthritis development. CD4$^+$ T cells (1 × 10^6) from Thy1.1$^+$ SKG mice were adoptively transferred into Thy1.2$^+$ *Rag2*$^{-/-}$ mice, which were i.v. injected with 500 μg anti-Thy1.2 mAb or control Rat IgG every week (n = 19 each). The severity of arthritis was monitored every week.

*$p < 0.05$, **$p < 0.01$. Data are representative of three independent experiments in (A)–(C), (E)–(J), (L), and (M) and pooled from more than two experiments in (D), (F), and (N). Vertical bars mean SD in (B), (E), (I), (K), (M), and (N).

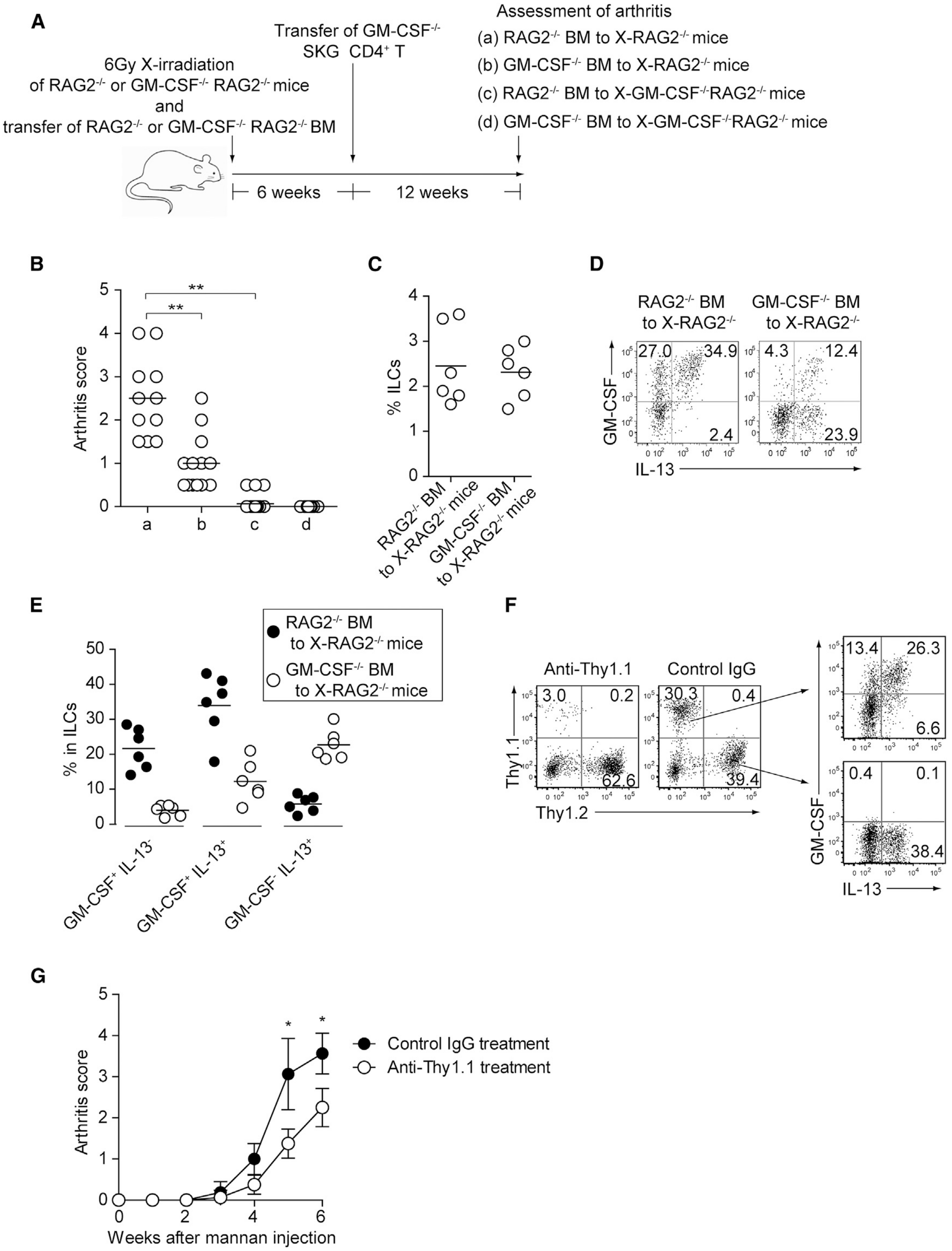

Figure 5. GM-CSF from ILCs and Radio-Resistant Stromal Cells Is Crucial for Autoimmune Arthritis

(A) Preparation of experimental groups for assessing arthritogenic effects of GM-CSF from ILCs or radio-resistant stromal cells. *Rag2*$^{-/-}$ or *Csf2*$^{-/-}$*Rag2*$^{-/-}$ mice were x-irradiated (X-*Rag2*$^{-/-}$ mice) and transferred with BM cells from *Csf2*$^{-/-}$ or WT *Rag2*$^{-/-}$ mice. The resulting four groups of BM chimeras were transferred with CD4^{+} T cells from *Csf2*$^{-/-}$ SKG mice 6 weeks after BM reconstitution and assessed for arthritis development 12 weeks later.

(B) Arthritis scores of four groups of mice shown in (A).

(C) Proportion of total synovial ILCs from x-irradiated *Rag2*−/− mice reconstituted with *Csf2*$^{-/-}$ or WT *Rag2*$^{-/-}$ BM cells.

(D) Flow cytometry of synovial ILCs for the expression of GM-CSF and IL-13 in arthritic joints of BM chimeras shown in (C).

(E) Proportion of GM-CSF^{+}IL-13^{-}, GM-CSF^{+}IL-13^{+}, and GM-CSF^{-}IL-13^{+} synovial ILCs (n = 6 each) as shown in (D).

(legend continued on next page)

Csf2$^{-/-}$ ILCs, which appeared to be hardly pathogenic (Figures 5D and 5E).

To further assess the contribution of ILCs to arthritis development, we x-irradiated Thy1.1^{+} *Rag2*$^{-/-}$ mice, reconstituted them with BM cells from Thy1.1^{+} *Rag2*$^{-/-}$ mice and Thy1.2^{+} *Csf2*$^{-/-}$ SKG mice at a 1:1 ratio, and treated the mice 6 weeks later with a single injection of 20 mg mannan and subsequently with 500 μg anti-Thy1.1 mAb once a week. Anti-Thy-1.1 treatment selectively depleted synovial Thy1.1^{+} GM-CSF-producing ILCs, while control mAb treatment allowed similar expansion of synovial Thy1.1^{+} GM-CSF-producing ILCs and Thy1.2^{+} GM-CSF-nonproducing ILCs (Figure 5F). This selective depletion of synovial GM-CSF-producing ILCs significantly reduced the severity of arthritis (Figure 5G).

These results collectively indicate that GM-CSF from both ILCs and radio-resistant stromal cells synergistically contributes to the development of severe autoimmune arthritis in SKG mice.

IL-2, IL-33, and TLR-9 Ligands Control GM-CSF Production by Synovial ILCs

Given the finding that IL-2, IL-7, and IL-33 control the effector function of ILC2s in inflamed tissues (Klose and Artis, 2016), we assessed their contribution to GM-CSF production by ILCs. Among various tissue homogenates prepared from normal mice, joint tissues substantially expressed IL-33, which significantly increased in arthritic joints at both the mRNA and protein levels compared with unaffected joints (Figures 6A and 6B). This was consistent with the finding by others that IL-33 was constitutively expressed in the nucleus of some immune cells and stromal cells in inflamed joints (Fock et al., 2013; Kaieda et al., 2010). We next sorted synovial ILCs and treated them *in vitro* with IL-2, IL-7, or IL-33 alone or in combination to assess the effects of these cytokines on GM-CSF production by ILCs. Intriguingly, IL-2, but not IL-7, in combination with IL-33 synergistically upregulated GM-CSF production from synovial ILCs, while the IL-2 and IL-33 combination or the IL-7 and IL-33 combination significantly increased the ILC2 signature cytokines IL-13 and IL-5 (Figure 6C). We also examined possible expression of TLRs by synovial ILCs, as reported with human ILCs in the tonsil and the intestine (Crellin et al., 2010; Marafini et al., 2015). Quantitative RT-PCR analysis revealed that synovial ILCs highly expressed *Tlr2*, *Tlr3*, *Tlr4*, and *Tlr9* relative to naive Th cells as a negative control (Figure 6D). Moreover, *in vitro* stimulation of synovial ILCs with TLR ligands alone or in combination with IL-33 showed that stimulation with each TLR ligand alone failed to induce any GM-CSF production, but CpG DNA, a TLR-9 ligand, in combination with IL-33 significantly increased GM-CSF production more than IL-33 alone (Figure 6E). Taken together, IL-33, IL-2, and TLR-9 ligands, especially their combination, in inflamed synovia are able to augment GM-CSF production by synovial ILCs.

GM-CSF-Producing ILCs in Synovial Fluid of RA Patients

With the key role of ILC-derived GM-CSF for the development of arthritis in SKG mice, we next evaluated the proportion and cytokine profile of ILCs in peripheral blood and synovial fluid (SF) obtained from RA or osteoarthritis (OA) patients. All the RA patients examined fulfilled the RA classification criteria of the American College of Rheumatology (ACR) and the European League Against Rheumatism (EULAR) 2010. The patients were 67.9 ± 8.9 (mean ± SD) years old, with disease duration of 25.4 ± 11.8 years. Anti-CCP antibody and rheumatoid factor were positive in all the patients. Disease activity scores in 28 joints using erythrocyte sedimentation rate (DAS28-ESR) were 3.0 ± 0.5. Among 10 patients, 7 were treated with methotrexate, 6 with biological or targeted synthetic disease-modifying anti-rheumatic drugs (tocilizumab; 2, etanercept; 1, golimumab; 1, abatacept; 1, tofacitinib; 1), and 9 with glucocorticoids (4.6 ± 2.1 mg/day prednisolone). The patients with OA were 77.7 ± 4.5 (mean ± SD) years old and fulfilled the grade 4 of Kellgren-Lawrence system for classification of OA. None of the OA patients were treated with immunosuppressive drugs or glucocorticoids. ILCs comprised a sizable fraction (~3%) of CD45^{+} hematopoietic cellular elements in SF from RA patients and were significantly increased when compared with ILCs in the peripheral blood of RA patients or those in SF from OA patients (Figures 7A, S2B, and S2C for gating strategies). Notably, the total numbers of SF ILCs from RA patients were markedly increased by ~100-fold compared with those from OA patients (Figure 7B). The ratio of ILCs expressing GM-CSF, IFN-γ, or IL-17 significantly increased, in RA SF compared with RA peripheral blood, with a significant reduction of the ratio of IL-13-expressing SF ILCs, although the latter appeared to increase in absolute number in RF SF compared with OA SF (Figure 7C). The majority of GM-CSF-producing cells in SF was CD3^{+} T cells (~80%), followed by CD11b^{+} myeloid cells (~15%) and ILCs (~5%) (Figures 7D and 7E). Thus, GM-CSF-producing synovial ILCs greatly expanded in inflamed joints of RA patients and shared a similar inflammatory signature between mice and humans. The difference in the cytokine expression profile, especially in IFN-γ and IL-13 expression, and in the cellular frequencies between human and mouse synovial ILCs could be attributed, at least in part, to the methods of sample collections (synovial tissues in mice versus SF in humans) as well as medications in the patients, different disease courses and stages, variable treatment periods, heterogeneity of disease types, and heterogenous genetic backgrounds of humans.

DISCUSSION

The main finding in this report is that arthritogenic Th17 cells are able to stimulate via IL-17 radio-resistant stromal cells including FLSs to secrete GM-CSF, and also synovial-resident ILCs to expand and produce GM-CSF in inflamed joints.

(F and G) The effects of ILC depletion on arthritis development. Thy1.1^{+} *Rag2*$^{-/-}$ mice were x-irradiated and transferred with BM cells from Thy1.1^{+} *Rag2*$^{-/-}$ and Thy1.2^{+} *Csf2*$^{-/-}$ SKG mice. The resulting BM chimeras were i.p. injected with 20 mg mannan 6 weeks later, followed by i.v. injection with 500 μg anti-Thy1.1 mAb or control Rat IgG every week. Flow cytometry of synovial ILCs for GM-CSF and IL-13 expression (F). Arthritis scores in each group of mice monitored every week (G). Vertical bars mean SD (n = 8 each).

*$p < 0.05$, **$p < 0.01$. Data are representative of three independent experiments in (D) and (F), and pooled from three experiments in (B), (C), (E), and (G). Horizontal bars indicate the means in (B), (C), and (E).

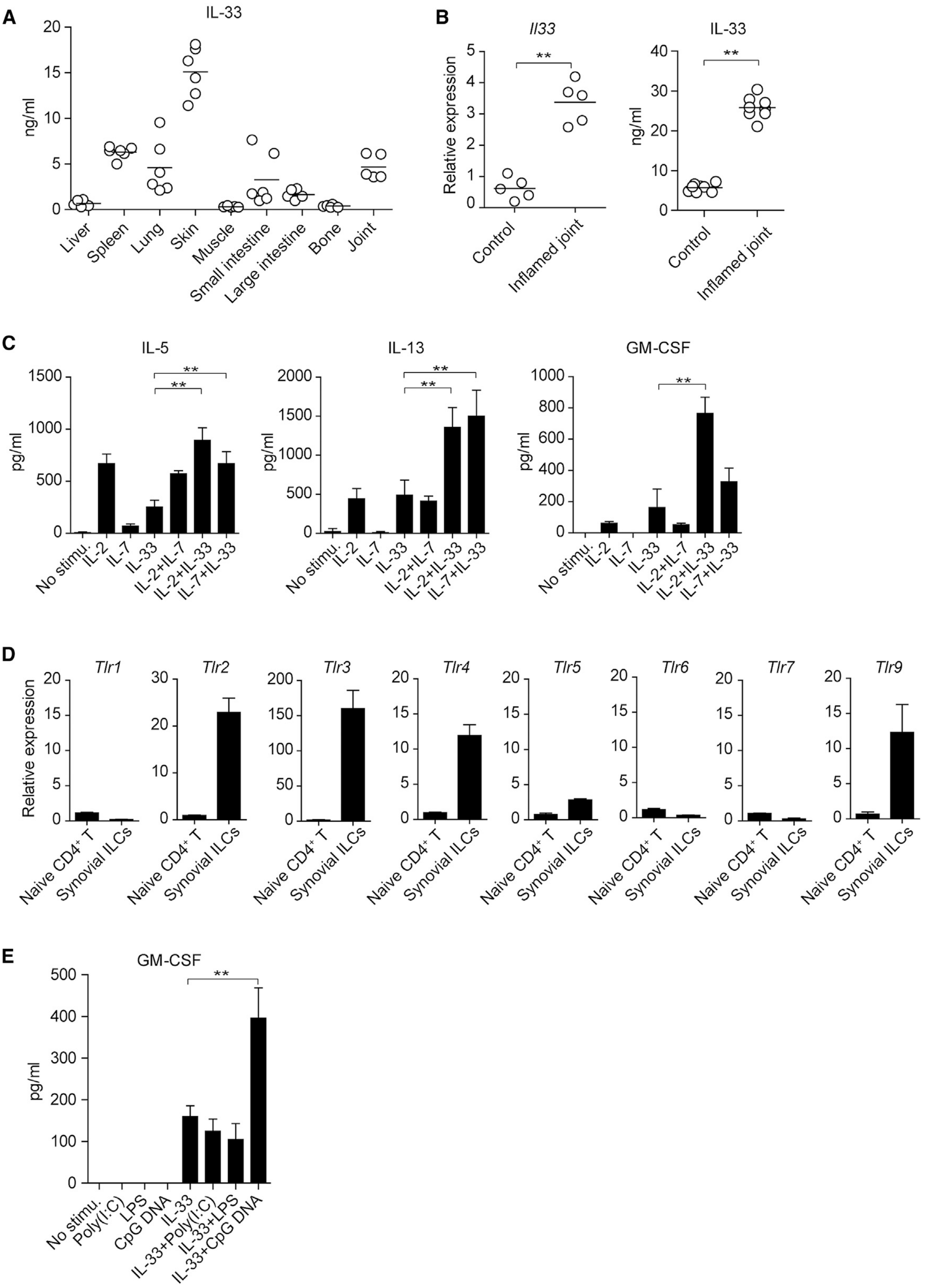

Figure 6. Control of GM-CSF Production in Synovial ILCs by IL-2, IL-33, and TLR-9 Ligands

(A) Quantity of the active form IL-33 assessed by ELISA (Biolegend, Mouse IL-33 ELISA kit) in indicated tissue homogenates.

(B) Quantitative RT-PCR for *Il33* expression and ELISA for IL-33 protein in arthritic or control joints. Symbols represent individual mice.

(C) Production of cytokines by synovial ILCs. Synovial ILCs (5×10^3) from arthritic joints were purified and cultured for 24 hr with rhIL-2 (20 U/mL), rmIL-7 (20 ng/mL), and rmIL-33 (20 ng/mL) alone or in combination. The concentration of IL-5, IL-13, and GM-CSF in the supernatant was measured (n = 3).

(legend continued on next page)

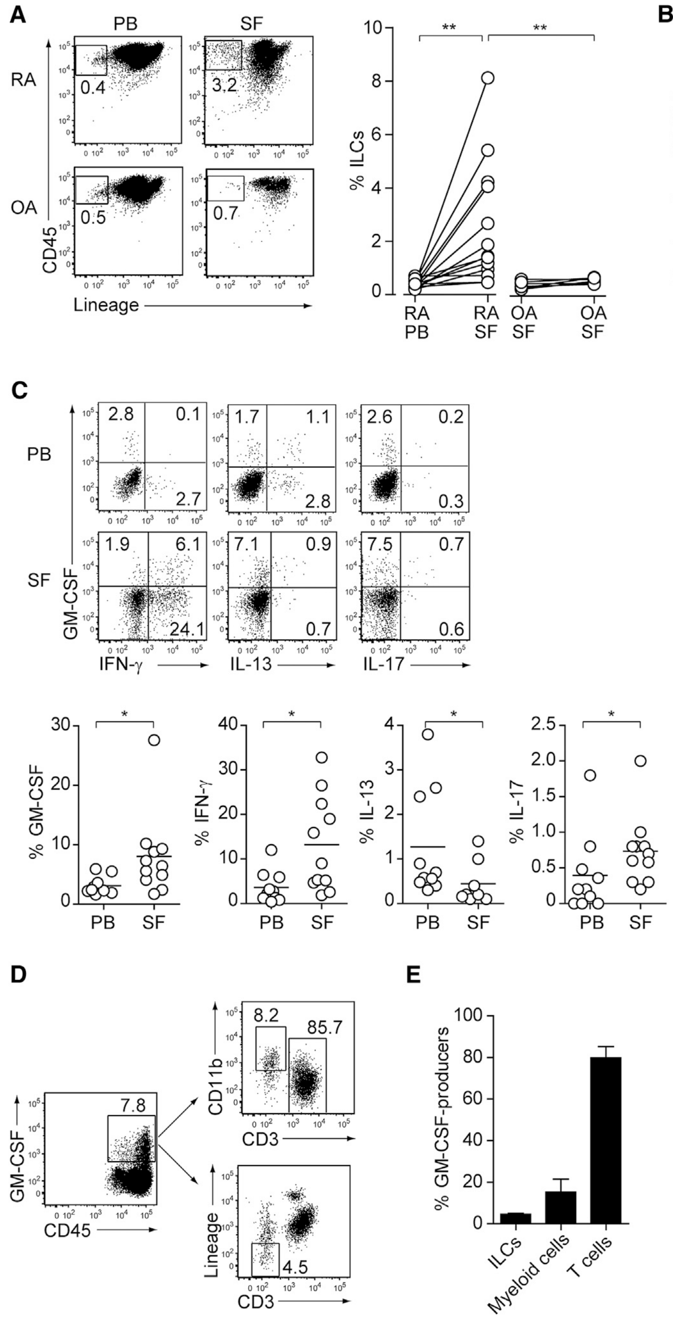

Figure 7. GM-CSF-Producing ILCs in Synovial Fluid of RA Patients

(A) The presence of ILCs (defined as $CD45^+$ $CD3^-CD4^-CD8^-CD11b^-CD11c^-CD19^-CD56^-$) from peripheral blood (PB) or synovial fluid (SF) of a patient with RA or OA (left). The percentages of ILCs in PB and SF from individual RA (n = 13) or OA (n = 6) patients. The lines indicate the sample pairs of the same patients (right).

(B) Total numbers of ILCs in 1 mL of SF from OA and RA patients (n = 6). Vertical bars indicate SD.

(C) Flow cytometry analysis of IFN-γ, IL-13, IL-17, and GM-CSF expression by ILCs (gated as in A) in PB or SF of a RA patient (top). The percentages of cytokine-producing ILCs from individual RA patients (n = 11) (bottom).

(D) Gating strategies for $GM\text{-}CSF^+CD45^+$ lineage markers-negative (ILCs), $GM\text{-}CSF^+CD45^+CD3^-$ $CD11b^+$ (myeloid cells), and $GM\text{-}CSF^+CD45^+$ $CD11b^-CD3^+$ cells (T cells).

(E) Proportion of GM-CSF-producing cells (n = 3). Vertical bars indicate SD. Symbols represent individual samples. Horizontal bars indicate the means.

*$p < 0.05$, **$p < 0.01$.

Abrogation of GM-CSF production by either the stromal cells or ILCs is able to prevent Th17 cell-mediated arthritis, while GM-CSF from arthritogenic Th cells is contributory to but not mandatory for arthritis development, at variance with a crucial role of Th17 cell-derived GM-CSF in mouse EAE (Codarri et al., 2011; El-Behi et al., 2011). The present results do not exclude possible contribution of GM-CSF-producing radio-resistant lymphocytes and non-lymphoid stromal cells other than ILCs and FLSs, inside or outside the joint, to disease manifestation in SKG mice and BM chimeras. Nevertheless, these results strongly support the notion that GM-CSF-producing radio-resistant stromal cells and synovial ILCs are critical components of a key inflammatory cellular cascade for the development of autoimmune arthritis.

It has been well substantiated in RA that FLSs in the synovial intimal lining are key effector cells producing large amounts of inflammatory mediators and proteolytic enzymes, such as matrix metalloproteinases (MMPs) and cathepsins, which degrade cartilage and bone (Bartok and Firestein, 2010). IL-1 and TNF-α synergistically induce GM-CSF in FLSs (Alvaro-Gracia et al., 1991). Stimulation with IL-17 in combination with IL-1 and TNF-α enhances the production of IL-6 and MMPs by FLSs (Chabaud et al., 2001). Here, we have shown that *in vitro* stimulation of SKG FLSs with IL-17 increases FLS-derived production of neutrophil-recruiting chemokines and GM-CSF, as previously reported with RA synoviocytes (Fossiez et al., 1996; Varas et al., 2015) and that loss of GM-CSF production by radio-resistant stromal cells including FLSs prevents arthritis development.

(D) Quantitative RT-PCR analysis of the expression of indicated TLR genes in naive $CD4^+$ T cells and synovial ILCs as shown in Figure 4M (n = 3).

(E) GM-CSF production by synovial ILCs. Synovial ILCs (5×10^3) were cultured for 24 hr with poly(I:C) (1 μg/mL), LPS (1 μg/mL), and CpG DNA (1 μM) alone or in combination with rmIL-33 (20 ng/mL). The concentration of GM-CSF in the supernatant was measured (n = 3).

**$p < 0.01$. Data are representative of at least two independent experiments. Horizontal bars indicate the means in (A) and (B). Vertical bars indicate SD in (C)–(E).

Since the induction of arthritis is dependent on IL-17 from autoimmune Th cells in our model (Hirota et al., 2007a), it is likely that Th17 cells stimulated the stromal cells via IL-17 to produce GM-CSF. This can be a key early event to initiate micro-inflammation, which in turn mediates a downstream inflammatory cascade, leading to overt joint inflammation accompanying the infiltration and expansion of Th17 cells, ILCs, neutrophils, and inflammatory monocytes.

GM-CSF-producing synovial-resident ILCs expand in inflamed joints and augment autoimmune arthritis. Among group 1, 2, and 3 ILCs, which are classified by their expression of signature cytokines and master transcription factors (Buonocore et al., 2010; Eberl et al., 2015; Spits et al., 2013), ILC2s mediate type 2 immunity including allergic reactions and host defense against parasite infections (Moro et al., 2010; Neill et al., 2010; Saenz et al., 2010; Wilhelm et al., 2011). We have shown here that $CD25^{+}IL\text{-}33Ra^{+}GATA\text{-}3^{+}$ ILC2s actively secreting GM-CSF are a major ILC subset expanding in inflamed joints and that depletion of total ILCs by anti-Thy1 antibody or loss of GM-CSF production in ILCs, without affecting the expansion of GM-CSF-nonproducing synovial ILCs, is able to effectively prevent autoimmune arthritis. Notably, these GM-CSF-producing synovial ILCs are physiologically present in healthy joints of any mouse strain. In addition, the presence of synovial ILCs in healthy joints and the paucity of ILCs in lymphoid organs or blood (Bando et al., 2015; Huang et al., 2015) indicate that ILC's precursors with the capacity to give rise to ILC1s, ILC2s, and ILC3s preferentially migrate from the BM, their primary site of generation, and differentiate into GM-CSF-producing ILCs in the synovia. While bidirectional plasticity between ILC1s and ILC3s was recently observed in the intestinal lamina propria (Bernink et al., 2015), synovial ILC2s did not appear to originate from ILC3s, which is the major GM-CSF producer in the intestine of mice and humans (Glatzer et al., 2013; Mortha et al., 2014). These results collectively suggest that GM-CSF may not be an ILC subset-specific cytokine but can be produced by ILC1s, ILC2s, and ILC3s in an environment-dependent manner, as a recent comprehensive transcriptome analysis of ILC subsets showed *Csf2* as one of shared transcriptional signatures (Robinette et al., 2015). In addition, Bhlhe40, which transcriptionally increases the expression of proinflammatory cytokines including GM-CSF in Th1 and Th17 cells (Lin et al., 2014; Martínez-Llordella et al., 2013), may be another shared molecule driving *Csf2* among ILC subsets and may also induce the pathogenicity of GM-CSF-producing synovial ILCs in autoimmune arthritis. It remains to be determined how such synovial ILCs are maintained as a cellular constituent of the normal joint and what physiological roles GM-CSF-producing synovial ILCs play in healthy joints.

GM-CSF production by ILCs is stimulated by the alarmin IL-33 and further enhanced by IL-2 and TLR-9 ligands. Synovial ILCs highly expressed CD25 and IL-33Ra, enabling their ligands IL-2 and IL-33, which are abundantly present in inflamed joints, to control GM-CSF production. In addition to well-characterized IL-2- and IL-33-dependent ILC2 control, we have shown in the current study that synovial ILCs expressed functional cytoplasmic TLR9, which may sense mitochondrial DNA (containing unmethylated CpG repeats, a bacterial molecular motif) possibly released as an endogenous damage-associated molecular pattern (DAMPs) (Zhang et al., 2010). In addition, extracellular mitochondrial DNA, which could experimentally induce arthritis in animals, was reported to be detectable in synovial fluid of RA patients (Collins et al., 2004). Taken together, synovial ILCs expressing CD25, IL-33Ra, and cytoplasmic TLR9 may sense IL-2, IL-33, and self DNA, which are produced by pathogenic Th cells or released from necrotic joint inflammatory cells, leading to their production of GM-CSF (Xu et al., 2008).

GM-CSF is abundant in RA synovium and upregulated by IL-1 and TNF-α and by IL-17 (Alvaro-Gracia et al., 1991; Varas et al., 2015). Anti-GM-CSFRα or anti-GM-CSF showed significant clinical efficacy in RA patients without major adverse effects such as pulmonary alveolar proteinosis, which is caused by autoantibodies against GM-CSF (Behrens et al., 2015; Burmester et al., 2013). As GM-CSF-producing ILCs, stromal cells, and T cells sense different inflammatory cues such as DAMPs, inflammatory cytokines, and recognition of self-antigens, respectively, in joint inflammation, the importance of each source of GM-CSF may depend on the phase of joint inflammation. For example, GM-CSF from ILCs appears to play a key role in the initiation of autoimmune arthritis, while T cell-derived GM-CSF may contribute to chronic progression of the disease. For better control of local tissue inflammation in RA and other immunological diseases, it is required to further elucidate how GM-CSF production is controlled in T cells, FLSs, and ILCs at various inflammation stages, and how these cell populations interact spatio-temporarily via GM-CSF.

STAR★METHODS

Detailed methods are provided in the online version of this paper and include the following:

- KEY RESOURCES TABLE
- CONTACT FOR REAGENT AND RESOURCE SHARING
- EXPERIMENTAL MODEL AND SUBJECT DETAILS
  - Mice
  - Induction of autoimmune arthritis
  - Patient information
- METHOD DETAILS
  - Flow cytometry
  - Preparation of FLS from inflamed joints
  - Quantitative RT-PCR
  - Culture and cytokine measurement of synovial ILCs
- QUANTIFICATION AND STATISTICAL ANALYSIS

SUPPLEMENTAL INFORMATION

Supplemental Information includes four figures and can be found with this article online at https://doi.org/10.1016/j.immuni.2018.04.009.

ACKNOWLEDGMENTS

This work was supported by grants-in-aid from the Ministry of Education, Culture, Sports, Science and Technology of Japan (16H06233 and 17K19553 to K.H. and 16H06295 to S.S.), the Core Research for Evolutional Science and Technology (CREST) program from the Japan Science and Technology Agency (S.S.), and Takeda Science Foundation (K.H.). We would like to thank

R. Ishii for technical assistance and the staff of the animal facility for breeding and maintenance of our mouse strains.

AUTHOR CONTRIBUTIONS

Conceptualization, K.H. and S.S.; Investigation, K.H., M.H., Y.I., M.M., A.T., K.Y., and N.S.; Writing – Original Draft, K.H. and S.S.; Funding Acquisition, K.H. and S.S.; Resources, H.I., M.T., H.W., G.K., M.K., A.J.P., and B.S.; Supervision, K.H. and S.S.

DECLARATION OF INTERESTS

The authors declare no competing interests.

Received: November 19, 2016
Revised: February 28, 2018
Accepted: April 6, 2018
Published: May 22, 2018

REFERENCES

Alvaro-Gracia, J.M., Zvaifler, N.J., and Firestein, G.S. (1989). Cytokines in chronic inflammatory arthritis. IV. Granulocyte/macrophage colony-stimulating factor-mediated induction of class II MHC antigen on human monocytes: a possible role in rheumatoid arthritis. J. Exp. Med. *170*, 865–875.

Alvaro-Gracia, J.M., Zvaifler, N.J., Brown, C.B., Kaushansky, K., and Firestein, G.S. (1991). Cytokines in chronic inflammatory arthritis. VI. Analysis of the synovial cells involved in granulocyte-macrophage colony-stimulating factor production and gene expression in rheumatoid arthritis and its regulation by IL-1 and tumor necrosis factor-alpha. J. Immunol. *146*, 3365–3371.

Bando, J.K., Liang, H.E., and Locksley, R.M. (2015). Identification and distribution of developing innate lymphoid cells in the fetal mouse intestine. Nat. Immunol. *16*, 153–160.

Bartok, B., and Firestein, G.S. (2010). Fibroblast-like synoviocytes: key effector cells in rheumatoid arthritis. Immunol. Rev. *233*, 233–255.

Behrens, F., Tak, P.P., Østergaard, M., Stoilov, R., Wiland, P., Huizinga, T.W., Berenfus, V.Y., Vladeva, S., Rech, J., Rubbert-Roth, A., et al. (2015). MOR103, a human monoclonal antibody to granulocyte-macrophage colony-stimulating factor, in the treatment of patients with moderate rheumatoid arthritis: results of a phase Ib/IIa randomised, double-blind, placebo-controlled, dose-escalation trial. Ann. Rheum. Dis. *74*, 1058–1064.

Bernink, J.H., Krabbendam, L., Germar, K., de Jong, E., Gronke, K., Kofoed-Nielsen, M., Munneke, J.M., Hazenberg, M.D., Villaudy, J., Buskens, C.J., et al. (2015). Interleukin-12 and -23 control plasticity of CD127(+) group 1 and group 3 innate lymphoid cells in the intestinal lamina propria. Immunity *43*, 146–160.

Buonocore, S., Ahern, P.P., Uhlig, H.H., Ivanov, I.I., Littman, D.R., Maloy, K.J., and Powrie, F. (2010). Innate lymphoid cells drive interleukin-23-dependent innate intestinal pathology. Nature *464*, 1371–1375.

Burmester, G.R., Weinblatt, M.E., McInnes, I.B., Porter, D., Barbarash, O., Vatutin, M., Szombati, I., Esfandiari, E., Sleeman, M.A., Kane, C.D., et al.; EARTH Study Group (2013). Efficacy and safety of mavrilimumab in subjects with rheumatoid arthritis. Ann. Rheum. Dis. *72*, 1445–1452.

Chabaud, M., Page, G., and Miossec, P. (2001). Enhancing effect of IL-1, IL-17, and TNF-alpha on macrophage inflammatory protein-3alpha production in rheumatoid arthritis: regulation by soluble receptors and Th2 cytokines. J. Immunol. *167*, 6015–6020.

Cho, J.H., and Feldman, M. (2015). Heterogeneity of autoimmune diseases: pathophysiologic insights from genetics and implications for new therapies. Nat. Med. *21*, 730–738.

Codarri, L., Gyülvészi, G., Tosevski, V., Hesske, L., Fontana, A., Magnenat, L., Suter, T., and Becher, B. (2011). RORγt drives production of the cytokine GM-CSF in helper T cells, which is essential for the effector phase of autoimmune neuroinflammation. Nat. Immunol. *12*, 560–567.

Collins, L.V., Hajizadeh, S., Holme, E., Jonsson, I.M., and Tarkowski, A. (2004). Endogenously oxidized mitochondrial DNA induces in vivo and in vitro inflammatory responses. J. Leukoc. Biol. *75*, 995–1000.

Crellin, N.K., Trifari, S., Kaplan, C.D., Satoh-Takayama, N., Di Santo, J.P., and Spits, H. (2010). Regulation of cytokine secretion in human CD127(+) LTi-like innate lymphoid cells by Toll-like receptor 2. Immunity *33*, 752–764.

Croxford, A.L., Lanzinger, M., Hartmann, F.J., Schreiner, B., Mair, F., Pelczar, P., Clausen, B.E., Jung, S., Greter, M., and Becher, B. (2015). The cytokine GM-CSF drives the inflammatory signature of CCR2+ monocytes and licenses autoimmunity. Immunity *43*, 502–514.

Eberl, G., Colonna, M., Di Santo, J.P., and McKenzie, A.N. (2015). Innate lymphoid cells. Innate lymphoid cells: a new paradigm in immunology. Science *348*, aaa6566.

El-Behi, M., Ciric, B., Dai, H., Yan, Y., Cullimore, M., Safavi, F., Zhang, G.X., Dittel, B.N., and Rostami, A. (2011). The encephalitogenicity of T(H)17 cells is dependent on IL-1- and IL-23-induced production of the cytokine GM-CSF. Nat. Immunol. *12*, 568–575.

Fock, V., Mairhofer, M., Otti, G.R., Hiden, U., Spittler, A., Zeisler, H., Fiala, C., Knöfler, M., and Pollheimer, J. (2013). Macrophage-derived IL-33 is a critical factor for placental growth. J. Immunol. *191*, 3734–3743.

Fossiez, F., Djossou, O., Chomarat, P., Flores-Romo, L., Ait-Yahia, S., Maat, C., Pin, J.J., Garrone, P., Garcia, E., Saeland, S., et al. (1996). T cell interleukin-17 induces stromal cells to produce proinflammatory and hematopoietic cytokines. J. Exp. Med. *183*, 2593–2603.

Glatzer, T., Killig, M., Meisig, J., Ommert, I., Luetke-Eversloh, M., Babic, M., Paclik, D., Blüthgen, N., Seidl, R., Seifarth, C., et al. (2013). RORγt^{+} innate lymphoid cells acquire a proinflammatory program upon engagement of the activating receptor NKp44. Immunity *38*, 1223–1235.

Hashimoto, M., Hirota, K., Yoshitomi, H., Maeda, S., Teradaira, S., Akizuki, S., Prieto-Martin, P., Nomura, T., Sakaguchi, N., Köhl, J., et al. (2010). Complement drives Th17 cell differentiation and triggers autoimmune arthritis. J. Exp. Med. *207*, 1135–1143.

Hata, H., Sakaguchi, N., Yoshitomi, H., Iwakura, Y., Sekikawa, K., Azuma, Y., Kanai, C., Moriizumi, E., Nomura, T., Nakamura, T., and Sakaguchi, S. (2004). Distinct contribution of IL-6, TNF-alpha, IL-1, and IL-10 to T cell-mediated spontaneous autoimmune arthritis in mice. J. Clin. Invest. *114*, 582–588.

Hirota, K., Hashimoto, M., Yoshitomi, H., Tanaka, S., Nomura, T., Yamaguchi, T., Iwakura, Y., Sakaguchi, N., and Sakaguchi, S. (2007a). T cell self-reactivity forms a cytokine milieu for spontaneous development of IL-17+ Th cells that cause autoimmune arthritis. J. Exp. Med. *204*, 41–47.

Hirota, K., Yoshitomi, H., Hashimoto, M., Maeda, S., Teradaira, S., Sugimoto, N., Yamaguchi, T., Nomura, T., Ito, H., Nakamura, T., et al. (2007b). Preferential recruitment of CCR6-expressing Th17 cells to inflamed joints via CCL20 in rheumatoid arthritis and its animal model. J. Exp. Med. *204*, 2803–2812.

Hirota, K., Duarte, J.H., Veldhoen, M., Hornsby, E., Li, Y., Cua, D.J., Ahlfors, H., Wilhelm, C., Tolaini, M., Menzel, U., et al. (2011). Fate mapping of IL-17-producing T cells in inflammatory responses. Nat. Immunol. *12*, 255–263.

Hoyler, T., Klose, C.S., Souabni, A., Turqueti-Neves, A., Pfeifer, D., Rawlins, E.L., Voehringer, D., Busslinger, M., and Diefenbach, A. (2012). The transcription factor GATA-3 controls cell fate and maintenance of type 2 innate lymphoid cells. Immunity *37*, 634–648.

Huang, Y., Guo, L., Qiu, J., Chen, X., Hu-Li, J., Siebenlist, U., Williamson, P.R., Urban, J.F., Jr., and Paul, W.E. (2015). IL-25-responsive, lineage-negative KLRG1(hi) cells are multipotential 'inflammatory' type 2 innate lymphoid cells. Nat. Immunol. *16*, 161–169.

Ito, Y., Hashimoto, M., Hirota, K., Ohkura, N., Morikawa, H., Nishikawa, H., Tanaka, A., Furu, M., Ito, H., Fujii, T., et al. (2014). Detection of T cell responses to a ubiquitous cellular protein in autoimmune disease. Science *346*, 363–368.

Ivanov, I.I., McKenzie, B.S., Zhou, L., Tadokoro, C.E., Lepelley, A., Lafaille, J.J., Cua, D.J., and Littman, D.R. (2006). The orphan nuclear receptor RORgammat directs the differentiation program of proinflammatory IL-17+ T helper cells. Cell *126*, 1121–1133.

Iwakura, Y., Ishigame, H., Saijo, S., and Nakae, S. (2011). Functional specialization of interleukin-17 family members. Immunity *34*, 149–162.

Kaieda, S., Shin, K., Nigrovic, P.A., Seki, K., Lee, R.T., Stevens, R.L., and Lee, D.M. (2010). Synovial fibroblasts promote the expression and granule accumulation of tryptase via interleukin-33 and its receptor ST-2 (IL1RL1). J. Biol. Chem. *285*, 21478–21486.

Klose, C.S., and Artis, D. (2016). Innate lymphoid cells as regulators of immunity, inflammation and tissue homeostasis. Nat. Immunol. *17*, 765–774.

Lin, C.C., Bradstreet, T.R., Schwarzkopf, E.A., Sim, J., Carrero, J.A., Chou, C., Cook, L.E., Egawa, T., Taneja, R., Murphy, T.L., et al. (2014). Bhlhe40 controls cytokine production by T cells and is essential for pathogenicity in autoimmune neuroinflammation. Nat. Commun. *5*, 3551.

Marafini, I., Monteleone, I., Di Fusco, D., Cupi, M.L., Paoluzi, O.A., Colantoni, A., Ortenzi, A., Izzo, R., Vita, S., De Luca, E., et al. (2015). TNF-α producing innate lymphoid cells (ILCs) are increased in active celiac disease and contribute to promote intestinal atrophy in mice. PLoS ONE *10*, e0126291.

Martínez-Llordella, M., Esensten, J.H., Bailey-Bucktrout, S.L., Lipsky, R.H., Marini, A., Chen, J., Mughal, M., Mattson, M.P., Taub, D.D., and Bluestone, J.A. (2013). CD28-inducible transcription factor DEC1 is required for efficient autoreactive CD4+ T cell response. J. Exp. Med. *210*, 1603–1619.

McGeachy, M.J., Chen, Y., Tato, C.M., Laurence, A., Joyce-Shaikh, B., Blumenschein, W.M., McClanahan, T.K., O'Shea, J.J., and Cua, D.J. (2009). The interleukin 23 receptor is essential for the terminal differentiation of interleukin 17-producing effector T helper cells in vivo. Nat. Immunol. *10*, 314–324.

Moro, K., Yamada, T., Tanabe, M., Takeuchi, T., Ikawa, T., Kawamoto, H., Furusawa, J., Ohtani, M., Fujii, H., and Koyasu, S. (2010). Innate production of T(H)2 cytokines by adipose tissue-associated c-Kit(+)Sca-1(+) lymphoid cells. Nature *463*, 540–544.

Mortha, A., Chudnovskiy, A., Hashimoto, D., Bogunovic, M., Spencer, S.P., Belkaid, Y., and Merad, M. (2014). Microbiota-dependent crosstalk between macrophages and ILC3 promotes intestinal homeostasis. Science *343*, 1249288.

Neill, D.R., Wong, S.H., Bellosi, A., Flynn, R.J., Daly, M., Langford, T.K., Bucks, C., Kane, C.M., Fallon, P.G., Pannell, R., et al. (2010). Nuocytes represent a new innate effector leukocyte that mediates type-2 immunity. Nature *464*, 1367–1370.

Parkes, M., Cortes, A., van Heel, D.A., and Brown, M.A. (2013). Genetic insights into common pathways and complex relationships among immune-mediated diseases. Nat. Rev. Genet. *14*, 661–673.

Robinette, M.L., Fuchs, A., Cortez, V.S., Lee, J.S., Wang, Y., Durum, S.K., Gilfillan, S., and Colonna, M.; Immunological Genome Consortium (2015). Transcriptional programs define molecular characteristics of innate lymphoid cell classes and subsets. Nat. Immunol. *16*, 306–317.

Saenz, S.A., Siracusa, M.C., Perrigoue, J.G., Spencer, S.P., Urban, J.F., Jr., Tocker, J.E., Budelsky, A.L., Kleinschek, M.A., Kastelein, R.A., Kambayashi, T., et al. (2010). IL25 elicits a multipotent progenitor cell population that promotes T(H)2 cytokine responses. Nature *464*, 1362–1366.

Sakaguchi, N., Takahashi, T., Hata, H., Nomura, T., Tagami, T., Yamazaki, S., Sakihama, T., Matsutani, T., Negishi, I., Nakatsuru, S., and Sakaguchi, S. (2003). Altered thymic T-cell selection due to a mutation of the ZAP-70 gene causes autoimmune arthritis in mice. Nature *426*, 454–460.

Setoguchi, R., Hori, S., Takahashi, T., and Sakaguchi, S. (2005). Homeostatic maintenance of natural Foxp3(+) CD25(+) CD4(+) regulatory T cells by interleukin (IL)-2 and induction of autoimmune disease by IL-2 neutralization. J. Exp. Med. *201*, 723–735.

Sonderegger, I., Iezzi, G., Maier, R., Schmitz, N., Kurrer, M., and Kopf, M. (2008). GM-CSF mediates autoimmunity by enhancing IL-6-dependent Th17 cell development and survival. J. Exp. Med. *205*, 2281–2294.

Spits, H., Artis, D., Colonna, M., Diefenbach, A., Di Santo, J.P., Eberl, G., Koyasu, S., Locksley, R.M., McKenzie, A.N., Mebius, R.E., et al. (2013). Innate lymphoid cells–a proposal for uniform nomenclature. Nat. Rev. Immunol. *13*, 145–149.

Tanaka, S., Maeda, S., Hashimoto, M., Fujimori, C., Ito, Y., Teradaira, S., Hirota, K., Yoshitomi, H., Katakai, T., Shimizu, A., et al. (2010). Graded attenuation of TCR signaling elicits distinct autoimmune diseases by altering thymic T cell selection and regulatory T cell function. J. Immunol. *185*, 2295–2305.

Varas, A., Valencia, J., Lavocat, F., Martínez, V.G., Thiam, N.N., Hidalgo, L., Fernández-Sevilla, L.M., Sacedón, R., Vicente, A., and Miossec, P. (2015). Blockade of bone morphogenetic protein signaling potentiates the pro-inflammatory phenotype induced by interleukin-17 and tumor necrosis factor-α combination in rheumatoid synoviocytes. Arthritis Res. Ther. *17*, 192.

Veldhoen, M., Hocking, R.J., Atkins, C.J., Locksley, R.M., and Stockinger, B. (2006). TGFbeta in the context of an inflammatory cytokine milieu supports de novo differentiation of IL-17-producing T cells. Immunity *24*, 179–189.

Wilhelm, C., Hirota, K., Stieglitz, B., Van Snick, J., Tolaini, M., Lahl, K., Sparwasser, T., Helmby, H., and Stockinger, B. (2011). An IL-9 fate reporter demonstrates the induction of an innate IL-9 response in lung inflammation. Nat. Immunol. *12*, 1071–1077.

Xu, D., Jiang, H.R., Kewin, P., Li, Y., Mu, R., Fraser, A.R., Pitman, N., Kurowska-Stolarska, M., McKenzie, A.N., McInnes, I.B., and Liew, F.Y. (2008). IL-33 exacerbates antigen-induced arthritis by activating mast cells. Proc. Natl. Acad. Sci. USA *105*, 10913–10918.

Yoshitomi, H., Sakaguchi, N., Kobayashi, K., Brown, G.D., Tagami, T., Sakihama, T., Hirota, K., Tanaka, S., Nomura, T., Miki, I., et al. (2005). A role for fungal beta-glucans and their receptor Dectin-1 in the induction of autoimmune arthritis in genetically susceptible mice. J. Exp. Med. *201*, 949–960.

Zhang, Q., Raoof, M., Chen, Y., Sumi, Y., Sursal, T., Junger, W., Brohi, K., Itagaki, K., and Hauser, C.J. (2010). Circulating mitochondrial DAMPs cause inflammatory responses to injury. Nature *464*, 104–107.

STAR★METHODS

KEY RESOURCES TABLE

REAGENT or RESOURCE	SOURCE	IDENTIFIER
Antibodies		
Anti-mouse CCR6 (29-2L17)-PE	Biolegend	Cat#129803; RRID: AB_1279139
Anti-mouse CD3e (145-2C11)-Biotin	BD Biosciences	Cat#553060; RRID: AB_394593
Anti-mouse CD4 (RM4-4)-Biotin	Biolegend	Cat#116010; RRID: AB_2561504
Anti-mouse CD8 (53-6.7)-Biotin	Biolegend	Cat#100704; RRID: AB_312743
Anti-mouse/human CD11b (M1/70)-Biotin	Biolegend	Cat#101203; RRID: AB_312786
Anti-mouse CD11c (HL3)-Biotin	BD Biosciences	Cat#553800; RRID: AB_395059
Anti-mouse CD16/32 (2.4G2)	BD Biosciences	Cat#553142; RRID: AB_394657
Anti-mouse CD19 (1D3)-Biotin	BD Biosciences	Cat#553784; RRID: AB_395048
Anti-mouse CD25 (PC61)-PE	BD Biosciences	Cat#553866; RRID: AB_395101
Anti-mouse CD44 (IM7)-PE	BD Biosciences	Cat#553134; RRID: AB_39464
Anti-mouse CD45.2 (104)-APC	Biolegend	Cat#109814; RRID: AB_389211
Anti-mouse c-Kit (2B8)-PE	Biolegend	Cat#105807; RRID: AB_313216
Anti-mouse Foxp3 (FJK-16S)-APC	eBioscience	Cat#17-5773-82; RRID: AB_469457
Anti-mouse Gata-3 (TWAI)-PE	eBioscience	Cat#12-9966-42; RRID: AB_1963600
Anti-mouse GM-CSF (MP1-22E9)-PE	BD Biosciences	Cat#554406; RRID: AB_395371
Anti-mouse GM-CSFRa (698423)-APC	R&D systems	Cat#FAB6130A; RRID: AB_10973836
Anti-mouse IFN-γ (XMG1.2)-eF450	eBioscience	Cat#48-7311-82; RRID: AB_1834366
Anti-mouse IL-13 (eBio13A)-eF450	eBioscience	Cat#48-7133-82; RRID: AB_11219690
Anti-mouse IL-17 (TC11-18H10.1)-BV421	Biolegend	Cat#506926; RRID: AB_2632611
Anti-mouse IL-7Ra (SB/199)-PE	BD Biosciences	Cat#552543; RRID: AB_394417
Anti-mouse/human Ki-67 (MKI67)-PE	BD Biosciences	Cat#556027; RRID: AB_2266296
Anti-mouse IL-33Ra (D1H9)-PE	Biolegend	Cat#145304; RRID: AB_2561915
Anti-mouse Ly-6C (AL-21)-FITC	BD Biosciences	Cat#553104; RRID: AB_394628
Anti-mouse Ly-6G (1A8)-PE	Biolegend	Cat#127608; RRID: AB_1186099
Anti-mouse MHC2 (M5/114.15.2)-BV421	Biolegend	Cat#107631; RRID: AB_10900075
Anti-mouse Pan-NK (DX-5)-Biotin	Biolegend	Cat#108904; RRID: AB_313411
Anti-mouse Podoplanin (8.1.1)-APC	Biolegend	Cat#127410; RRID: AB_10613649
Anti-mouse Rorγt (AFKJS-9)-PE	eBioscience	Cat#12-6988-82; RRID: AB_1834470
Anti-mouse TCR-β (H57-597)-APC	Biolegend	Cat#109212; RRID: AB_313435
Anti-mouse T-bet (4B10)-BV421	Biolegend	Cat#644815; RRID: AB_10896427
Anti-mouse Thy1.1 (OX-7)-FITC	BD Biosciences	Cat#554897; RRID: AB_395588
Anti-mouse Thy1.2 (53-2.1)-FITC	Biolegend	Cat#140304; RRID: AB_10642812
Anti-mouse Thy1.2 (53-2.1)-BV421	Biolegend	Cat#140327; RRID: AB_2686992
Anti-human CD3 (UCHT1)-Biotin	Biolegend	Cat#300404; RRID: AB_314058
Anti-human CD3 (UCHT1)-Alexa488	Biolegend	Cat#300454; RRID: AB_2564149
Anti-human CD4 (OKT4)-Biotin	Biolegend	Cat#317406; RRID: AB_571949
Anti-human CD8 (SK1)-Biotin	Biolegend	Cat#344720; RRID: AB_2075392
Anti-mouse/human CD11b (M1/70)-PE	eBioscience	Cat#12-0112-81; RRID: AB_465549
Anti-human CD11c (3.9)-Biotin	Biolegend	Cat#301612; RRID: AB_493021
Anti-human CD19 (HIB19)-Biotin	Biolegend	Cat#302204; RRID: AB_314234
Anti-human CD45 (HI30)-BV421	Biolegend	Cat#304032; RRID: AB_2561357
Anti-human CD56 (HCD56)-Biotin	Biolegend	Cat#318320; RRID: AB_893390
Anti-human GM-CSF (BVD2-21C11)-APC	Biolegend	Cat#502310; RRID: AB_11150231
Anti-human IFN-γ (4S.B3)-Alexa488	Biolegend	Cat#502515; RRID: AB_493029

(*Continued on next page*)

Continued

REAGENT or RESOURCE	SOURCE	IDENTIFIER
Anti-human IL-13 (JES10-5A2)-PE	Biolegend	Cat#501903; RRID: AB_315198
Anti-human IL-17 (BL168)-Alexa488	Biolegend	Cat#512308; RRID: AB_961386
PECy7-Streptavidin	BD Biosciences	Cat#557598; RRID: AB_10049577
Anti-mouse Thy1.1 (19E12)	Bio X Cell	Cat#BE0214; RRID: AB_2687700
Anti-mouse Thy1.2 (30H12)	Bio X Cell	Cat#BE0066; RRID: AB_1107682
Isotype control Rat IgG2b (LTF-2)	Bio X Cell	Cat#BE0090; RRID: AB_1107780
Chemicals, Peptides, and Recombinant Proteins		
Mannan from Saccharomyces cerevisiae	Sigma-Aldrich	Cat#M7504
Phorbol 12-myristate 13-acetate	Sigma-Aldrich	Cat#P1585
Ionomycin	Sigma-Aldrich	Cat#I0634
Brefeldin A	Merck	Cat#203729
Liberase TM Research Grade	Roche	Cat#0540111901
TRIzol	Invitrogen	Cat#15596026
IMDM	Sigma-Aldrich	Cat#I3390-500ML
DMEM	Nacalai Tesque	Cat#08459-35
Penicillin-streptomycin solution	Nacalai Tesque	Cat#26252-94
37% Formaldehyde solution	Sigma-Aldrich	Cat#F8775-25ML
NP-40	Nacalai Tesque	Cat#23640-94
rhIL-2	Shionogi Co.	6399411D1022
rmIL-7	R&D systems	Cat#407-ML-005
rmIL-33	R&D systems	Cat#3626-ML-010
Poly(I:C)	InvivoGen	Cat#tlrl-picw
LPS	InvivoGen	Cat#tlrl-3pelps
CpGDNA	InvivoGen	Cat# tlrl-1585
FBS	GIBCO	Cat#10437-028
HBSS	Nacalai Tesque	Cat#17460-015
Sodium Azide	Nacalai Tesque	Cat#31208-82
Ficoll-Paque PLUS	GE Healthcare	Cat#17-1440-03
2-Mercaptoethanol	GIBCO	Cat#21985-023
Sodium pyruvate	GIBCO	Cat#11360-070
GlutaMAX	GIBCO	Cat#35050-61
MEM NEAA	GIBCO	Cat#11140-050
70 μm filter mesh	BD Biosciences	Cat#352350
Critical Commercial Assays		
Foxp3 Staining Buffer Set	eBioscience	Cat#00-5523-00
Mouse IL-33 ELISA Kit	Biolegend	Cat#436407
Arthrogen-CIA 5-Clone Cocktail Kit	Chondrex	Cat#53040
BD Cytometric Bead Array (IL-5)	BD Biosciences	Cat#558302
BD Cytometric Bead Array (IL-13)	BD Biosciences	Cat#558349
BD Cytometric Bead Array (GM-CSF)	BD Biosciences	Cat#558347
CD4 MicroBeads	Miltenyi Biotec	Cat#130-049-201
LS Column	Miltenyi Biotec	Cat#130-042-401
qPCR Master Mix	TOYOBO	Cat#QPS-101
SuperScript VILO Master Mix	Invitrogen	Cat#11756050
TaqMan Gene Expression (*Bhlhe40*)	ABI	Mm00478593_m1
TaqMan Gene Expression (*Ccl20*)	ABI	Mm01268754_m1
TaqMan Gene Expression (*Csf2*)	ABI	Mm00438328_m1
TaqMan Gene Expression (*Cxcl1*)	ABI	Mm04207460_m1
TaqMan Gene Expression (*Cxcl5*)	ABI	Mm00436451_g1

(*Continued on next page*)

Continued		
REAGENT or RESOURCE	SOURCE	IDENTIFIER
TaqMan Gene Expression (*Hprt*)	ABI	Mm01545399_m1
TaqMan Gene Expression (*Il1b*)	ABI	Mm00434228_m1
TaqMan Gene Expression (*Il6*)	ABI	Mm00446190_m1
TaqMan Gene Expression (*Il33*)	ABI	Mm00505403_m1
TaqMan Gene Expression (*Lif*)	ABI	Mm00434762_g1
TaqMan Gene Expression (*Nfkbiz*)	ABI	Mm00600522_m1
TaqMan Gene Expression (*Tlr1*)	ABI	Mm00446095_m1
TaqMan Gene Expression (*Tlr2*)	ABI	Mm00442346_m1
TaqMan Gene Expression (*Tlr3*)	ABI	Mm01207404_m1
TaqMan Gene Expression (*Tlr4*)	ABI	Mm00445273_m1
TaqMan Gene Expression (*Tlr5*)	ABI	Mm00546288_s1
TaqMan Gene Expression (*Tlr6*)	ABI	Mm02529782_s1
TaqMan Gene Expression (*Tlr7*)	ABI	Mm00446590_m1
TaqMan Gene Expression (*Tlr9*)	ABI	Mm00446193_m1
TaqMan Gene Expression (*Tnf*)	ABI	Mm00443260_g1
TaqMan Gene Expression (*Zc3h12a*)	ABI	Mm00462533_m1
Software and Algorithms		
FlowJo	Tree Star, Inc.	https://www.flowjo.com
GraphPad PRISM	GraphPad Software, Inc.	https://www.graphpad.com/
BD Cytometric Bead Array FCAP Array Software	BD Biosciences	Cat# 652099
Other		
BD FACS Canto II	BD Biosciences	http://www.bdbiosciences.com/us/instruments/research/cell-analyzers/bd-facscanto-ii/m/744810/features
BD FACS Aria SORP	BD Biosciences	N/A
Roche LightCycler 480	Roche	https://lifescience.roche.com/global_en/products/lightcycler14301-480-instrument-ii.html

CONTACT FOR REAGENT AND RESOURCE SHARING

Requests for data or reagents should be directed and will be fulfilled by the Lead Contact, Shimon Sakaguchi (shimon@ifrec.osaka-u.ac.jp).

EXPERIMENTAL MODEL AND SUBJECT DETAILS

Mice

SKG (Sakaguchi et al., 2003), *Il17a*$^{-/-}$ SKG (Hirota et al., 2007a), *Rag2*$^{-/-}$ (Setoguchi et al., 2005), *Csf2*$^{-/-}$ (Sonderegger et al., 2008), Thy1.1 congenic (Setoguchi et al., 2005) mice were used. C57/BL6J mice were purchased from CLEA Japan. *Il17a*^{Cre}, R26R^{FP635}, and R26R^{eYFP} mice were backcrossed to SKG for more than eight generations (Hirota et al., 2011). *Csf2*$^{-/-}$ SKG, *Csf2*$^{-/-}$ *Rag2*$^{-/-}$, *Il17a*^{Cre} R26R^{FP635}, *Il17a*^{Cre} R26R^{eYFP}, Thy1.1 congenic SKG or *Rag2*$^{-/-}$ mice were generated by crossing the strains described above. All the genetically modified mouse strains used in this study were on the BALB/c background and kept under specific pathogen-free conditions. Both male and female mice at 8-12 weeks of age were used for all the experiments. All animal experiments were approved by the Animal Ethical Committee of Immunology Frontier Research Center, Osaka University and Institute for Frontier Life and Medical Sciences, Kyoto University, and performed in accordance with institutional guidelines.

Induction of autoimmune arthritis

Arthritis was induced by either a single injection of 20 mg mannan (Sigma-Aldrich) intraperitoneally (Hashimoto et al., 2010), or adoptive transfer of CD4^{+} T cells intravenously (i.v.) (Hirota et al., 2007a). CD4^{+} T cells were sorted from the spleen and peripheral LNs of SKG mice using MACS CD4 microbeads and LS column (Miltenyi Biotec) according to the manufacturer's instruction. For depletion of ILCs, purified anti-Thy1.1 (19E12; Bio X cell), anti-Thy1.2 (30H12; Bio X Cell) or isotype control Rat IgG2b (LTF-2; Bio X Cell)

antibody was i.v. injected. Joint swelling was scored as follows: 0, no joint swelling; 0.1, swelling of one finger joint; 0.5, mild swelling of wrist or ankle; 1.0, severe swelling of wrist or ankle. Scores for all fingers of forepaws and hindpaws, wrists and ankles were totaled for each mouse.

Patient information

Detailed information about RA or OA patients is described in the section of Results. Synovial fluid samples from RA or OA patients were obtained at the time of arthrocentesis or surgical operation and cellular fractions were analyzed by flow cytometry. Samples from peripheral blood were taken from the same patients at different time points. Peripheral blood mononuclear cells were separated using Ficoll-Paque PLUS (GE Healthcare). This study has been approved by the Ethical Committee of Kyoto University. Written informed consent was obtained from all the participants.

METHOD DETAILS

Flow cytometry

The following monoclonal antibodies were used for flow cytometry analysis (BD FACSCantoII) and cell sorting (BD FACSAria SORP): anti-mouse CCR6 (29-2L17, Biolegend), CD3e (145-2C11, BD Biosciences), CD4 (RM4-4, Biolegend), CD8 (53-6.7, Biolegend), CD11b (M1/70, Biolegend), CD11c (HL3, BD Biosciences), CD16/32 (2.4G2, BD Biosciences), CD19 (1D3, BD Biosciences), CD25 (PC61, BD Biosciences), CD44 (IM7, BD Biosciences), CD45.2 (104, Biolegend), c-Kit (2B8, Biolegend), Foxp3 (FJK-16S, eBioscience), Gata-3 (TWAI, eBioscience), GM-CSF (MP1-22E9, BD Biosciences), GM-CSFRa (698423, R&D systems), IFN-γ (XMG1.2, eBioscience), IL-13 (eBio13A, eBioscience), IL-17 (TC11-18H10.1, Biolegend), IL-7Ra (SB/199, BD Biosciences), Ki-67 (MKI67, BD Biosciences), IL-33Ra (D1H9, Biolegend), Ly-6C (AL-21, BD Biosciences), Ly-6G (1A8, Biologend), MHC2 (M5/114.15.2, Biolegend), Pan-NK (DX-5, eBioscience), Podoplanin (8.1.1, Biolegend), Rorγt (AFKJS-9, eBioscience), TCR-β (H57-597, Biolegend), T-bet (4B10, Biolegend), Thy1.1 (OX-7, BD Biosciences), Thy1.2 (53-2.1, Biolegend), anti-human CD3 (UCHT1, Biolegend), CD4 (OKT4, Biolegend), CD8 (SK1, Biolegend), CD11b (M1/70, eBioscience), CD11c (3.9, Biolegend), CD19 (HIB19, Biolegend), CD45 (HI30, Biolegend), CD56 (HCD56, Biolegend), GM-CSF (BVD2-21C11, Biolegend), IFN-γ (4S.B3, Biolegend), IL-13 (JES10-5A2, Biolegend), IL-17 (BL168, Biolegend), PECy7-Streptavidin (BD Biosciences). For intracellular staining for transcription factors, cells were stained using Foxp3 staining buffer set (eBioscience) according to the manufacturer's instruction. For intracellular staining for cytokines, cells were restimulated in IMDM buffer (Sigma-Aldrich) supplemented with 5% FBS (GIBCO), penicillin-streptomycin (Nacalai Tesque), 2-Mercaptoethanol (GIBCO), GlutaMAX (GIBCO), sodium pyruvate (GIBCO), and MEM NEAA (GIBCO) for 4 h with phorbol 12-myristate 13-acetate (50 ng/ml; Sigma-Aldrich) and ionomycin (500 ng/ml; Sigma-Aldrich) in the presence of brefeldin A (1 μg/ml; Merck), fixed with 3.7% formaldehyde (Sigma-Aldrich), permeabilized with 0.1% NP-40 (Nacalai Tesque), and stained with FACS buffer consisting of HBSS (Nacalai Tesque) supplemented with 2% FBS (GIBCO) and 0.1% sodium azide (Nacalai Tesque). All the FACS data were analyzed on FlowJo software.

Preparation of FLS from inflamed joints

Inflammatory synovial cells were prepared by cutting the synovial tissues from arthritic joints into small pieces, followed by enzymatic digestion for 30 min at 37°C in plain IMDM buffer (Sigma-Aldrich) with Liberase TM (0.25 mg/ml; Roche), and then by mashing the digested tissues through 70-μm mesh filter (BD Biosciences). The resultant single cell suspension of synovial tissues was used for flow cytometry or cell sorting experiments. Non-hematopoietic synovial cells adherent to culture dishes were passaged several times without any stimulation in DMEM medium (Nacalai Tesque) supplemented with 20% FBS (GIBCO) and penicillin-streptomycin (Nacalai Tesque) in order to remove synovial immune cells and used as FLS.

Quantitative RT-PCR

Total RNA from FLS or inflammatory immune cells isolated from arthritic joints was extracted with TRIzol (Invitrogen) and reverse transcribed with SuperScript VILO (Invitrogen) in accordance with the manufacturer's instructions. The resultant cDNAs served as templates for the amplification of genes of interest and a housekeeping gene by LightCycler 480 (Roche) with qPCR Master Mix (TOYOBO) and ABI TaqMan Gene Expression assays (*Bhlhe40*; Mm00478593_m1, *Ccl20*; Mm01268754_m1, *Csf2*; Mm00438328_m1, *Cxcl1*; Mm04207460_m1, *Cxcl5*; Mm00436451_g1, *Hprt*; Mm01545399_m1, *Il1b*; Mm00434228_m1, *Il6*; Mm00446190_m1, *Il33*; Mm00505403_m1, *Lif*; Mm00434762_g1, *Nfkbiz*; Mm00600522_m1, *Tlr1*; Mm00446095_m1, *Tlr2*; Mm00442346_m1, *Tlr3*; Mm01207404_m1, *Tlr4*; Mm00445273_m1, *Tlr5*; Mm00546288_s1, *Tlr6*; Mm02529782_s1, *Tlr7*; Mm00446590_m1, *Tlr9*; Mm00446193_m1, *Tnf*; Mm00443260_g1, Zc3h12a; Mm00462533_m1). The level of target gene expression was quantified after normalization to *Hprt* expression.

Culture and cytokine measurement of synovial ILCs

Synovial ILCs ($CD3^-$, $CD4^-$, $CD8^-$, $CD11b^-$, $CD11c^-$, $CD19^-$, Pan-NK^-, $CD45.2^+$, and $Thy1.2^+$) from inflamed joints were purified by flow cytometry and cultured overnight in the presence of rhIL-2 (20 U/ml, Shionogi Co.), rmIL-7 (20 ng/ml, R&D systems), rmIL-33 (20 ng/ml, R&D systems), Poly(I:C) (1 μg/ml, InvivoGen), LPS (1 μg/ml, InvivoGen), and CpG DNA (1 μM, InvivoGen). The concentration of IL-5, IL-13, and GM-CSF in the culture supernatant was measured using BD Cytometric Bead Array (BD Biosciences).

QUANTIFICATION AND STATISTICAL ANALYSIS

Data are shown as mean ± standard error of mean (SEM) or mean ± standard deviation (SD). Statistical analysis was done with GraphPad PRISM. A two-tailed t test was used for statistical analysis. ANOVA and Bonferroni post-test were used for grouped data analysis. *P* value of < 0.05 was considered statistically significant. Sample sizes for all shown data can be found in the figure legends.

Immunity, Volume *48*

Supplemental Information

Autoimmune Th17 Cells Induced Synovial Stromal and Innate Lymphoid Cell Secretion of the Cytokine GM-CSF to Initiate and Augment Autoimmune Arthritis

Keiji Hirota, Motomu Hashimoto, Yoshinaga Ito, Mayumi Matsuura, Hiromu Ito, Masao Tanaka, Hitomi Watanabe, Gen Kondoh, Atsushi Tanaka, Keiko Yasuda, Manfred Kopf, Alexandre J. Potocnik, Brigitta Stockinger, Noriko Sakaguchi, and Shimon Sakaguchi

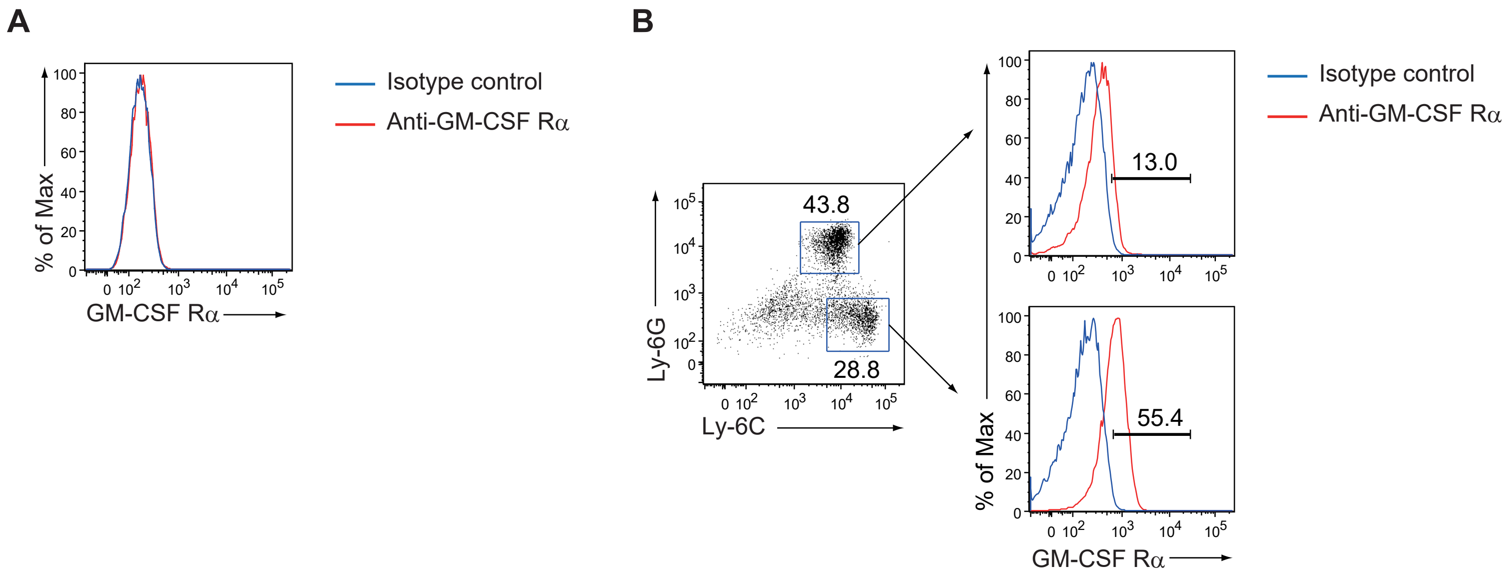

Figure S1. Flow cytometry of FLS and CD11b⁺ myeloid cells in inflamed joints, Related to Figure 3. **(A)** Staining for surface GM-CSF Rα of FLS isolated from SKG arthritic joints. **(B)** Surface GM-CSF Rα staining of $CD11b^+$ $Ly\text{-}6C^+$ $Ly\text{-}6G^-$ cells (inflammatory monocytes) and $CD11b^+$ $Ly\text{-}6C^+$ $Ly\text{-}6G^+$ cells (neutrophils) in SKG arthritic joints. Data are representative of two independent experiments.

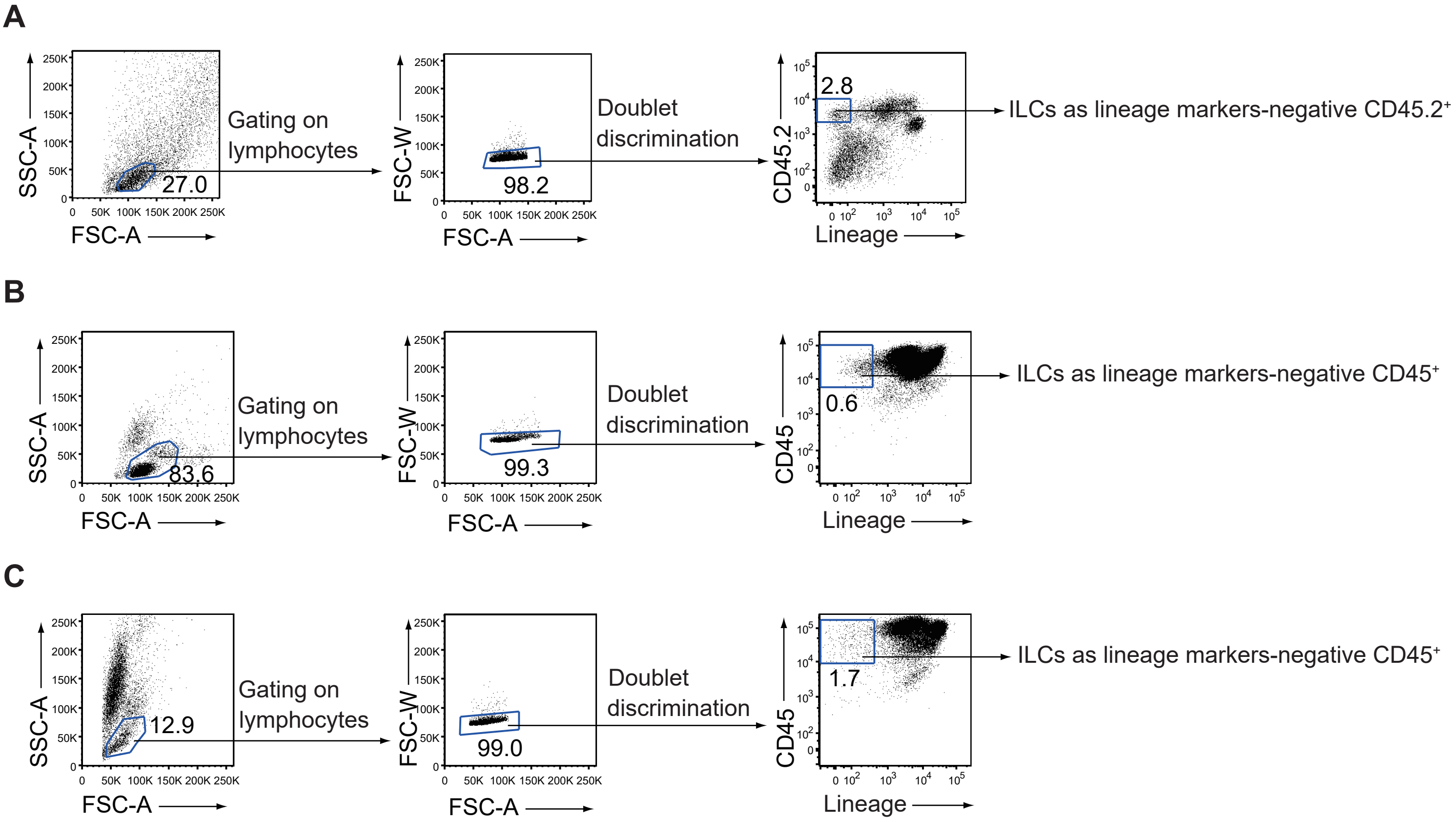

Figure S2. Gating strategies for mouse and human ILCs, Related to Figure 4 and Figure 7. Synovial cells from inflamed joints in mice **(A)**, peripheral blood **(B)** or synovial fluid **(C)** of RA patients were first gated on the lymphocyte fraction (left panel), then excluded of doublet cells using FSC-W (middle panel), and analyzed or purified as ILCs as CD45+ lineage markers-negative cells (right panel).

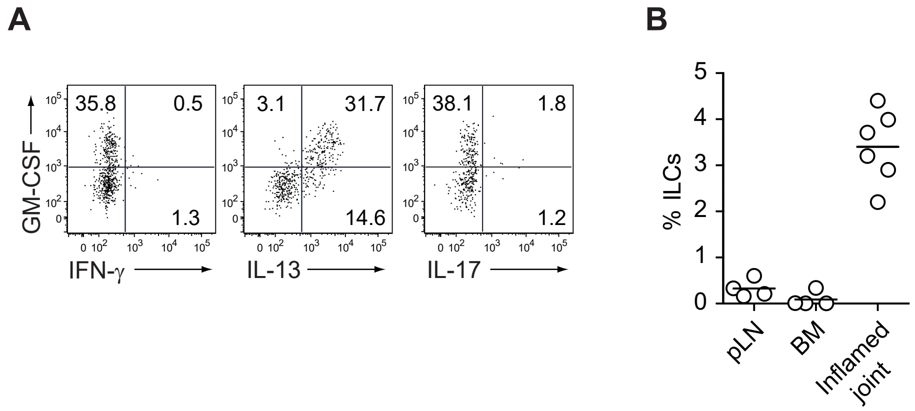

Figure S3. Flow cytometry of synovial ILCs in healthy joints of SKG mice, Related to Figure 4. **(A)** Flow cytometric analysis of intracellular GM-CSF, IFN-γ, IL-13, and IL-17 in ILCs from healthy joints of SKG mice. Data are representative of three independent experiments. **(B)** Proportion of ILCs in popliteal LNs (pLN), bone marrow (BM), and inflamed joints of SKG mice with severe arthritis induced by an injection of 20 mg mannan. Symbols represent individual mice. Horizontal lines indicate the means. Data are pooled from two independent experiments.

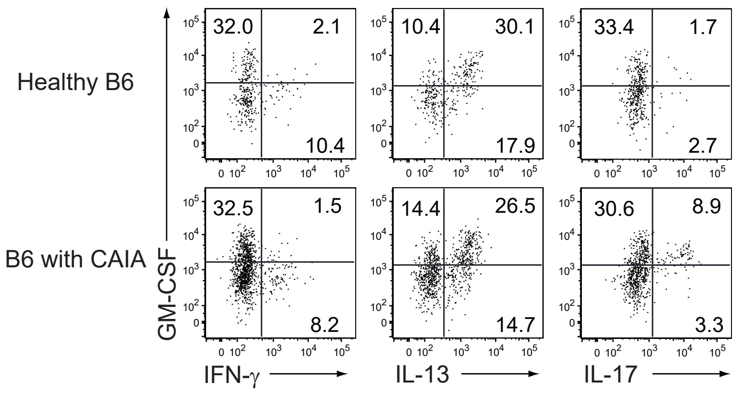

Figure S4. Flow cytometry of synovial ILCs in inflamed joints of C57/BL6 mice with collagen antibody-induced arthritis (CAIA), Related to Figure 4.

Flow cytometric analysis of intracellular GM-CSF, IFN-γ, IL-13, and IL-17 in ILCs from healthy or inflamed joints of C57/BL6 (B6) mice with CAIA. B6 mice were i.v. injected with a combination of monoclonal antibody cocktail (2 mg) specific for type II collagen (Arthrogen-CIA Arthritogenic Monoclonal Antibody, Chondrex, Inc.), i.p. injected with LPS (50 μg) three days later, and analyzed 2 weeks later. Data are representative of two independent experiments.
